# nNOS-mediated S-nitrosylation of TCOF1 regulates KRAS proteostasis to suppress hepatoblastoma progression

**DOI:** 10.1016/j.redox.2025.103870

**Published:** 2025-09-20

**Authors:** Meng Wang, Yupeng Wang, Yue Qian, Ziyan Luo, Siqi Dong, zhuoyan Li, Lingling Wu, Fang Yu, Zihua Lin, Lin Qiu, Hua Jiang, Linna Yu

**Affiliations:** a Department of Hematology and Oncology, Guangzhou Women and Children's Medical Center, Guangzhou Medical University, Guangzhou, 510623, China; b Department of Oncology, Zhujiang Hospital, Southern Medical University, Guangzhou, 510282, China; c Department of Ophthalmology, Guangzhou Women and Children's Medical Center, Guangzhou Medical University, Guangzhou, 510623, China; d Department of Emergency, Guangzhou Women and Children's Medical Center, Guangzhou Medical University, Guangzhou, 510623, China; e Department of Pharmacy, Guangdong Second Provincial Traditional Chinese Medicine Hospital, Guangzhou, 510095, China; f Department of Hematology and Oncology, Liuzhou Hospital, Guangzhou Women and Children's Medical Center, Liuzhou, 545616, China; g Department of Gastroenterology, The First People's Hospital of Yunnan Province, Kunming, 650032, China; h The Affiliated Hospital of Kunming University of Science and Technology, Kunming, 650032, China

**Keywords:** Hepatoblastoma, nNOS, KRAS, S-nitrosylation, TCOF1

## Abstract

Neuronal nitric oxide synthase (nNOS) plays dual roles in tumorigenesis, but its function in hepatoblastoma (HB) remains unclear. Analysis of 30 clinical HB samples reveals significant nNOS downregulation, correlating with tumor malignancy. Overexpression of nNOS inhibits HB cell proliferation and tumor growth in vitro and in vivo. Multi-omics analysis identifies the MAPK pathway as a key target, with KRAS protein levels most prominently reduced. Mechanistically, nNOS induces S-nitrosylation of TCOF1 at cysteine 644, disrupting TCOF1-KRAS interaction and thereby accelerating KRAS protein degradation. These findings establish the nNOS-TCOF1-KRAS axis as a critical regulator of HB progression and propose a novel NO-based therapeutic strategy for KRAS-driven cancers.

## 1 Introduction

Hepatoblastoma (HB), the predominant pediatric liver cancer, accounts for over 90 % of hepatic malignancies in children under five, with a global incidence rising by 4.2 % annually, surpassing most other childhood cancers [[1], [2], [3]]. Despite advances in multimodal therapies combining surgery, chemotherapy, and targeted agents, the prognosis for patients with advanced or refractory HB remains grim, with a 5-year overall survival (OS) rate of 52 % and a 1-year progression-free survival (PFS) rate below 24 % [[4], [5], [6]]. This poor prognosis is largely attributed to chemoresistance and uncontrolled tumor proliferation, which compromise current cisplatin-based neoadjuvant regimens in nearly 30 % of cases [7,8]. Elucidating the molecular drivers of HB proliferation is critical for advancing precision oncology, yet these drivers remain poorly defined [9,10].

Neuronal nitric oxide synthase (nNOS), an enzyme that generates nitric oxide (NO), exhibits contradictory roles across different cancers, underscoring the need for in-depth research into its tissue-specific mechanisms [[11], [12], [13], [14]]. In osteosarcoma, dysregulated nNOS activity causes BH4 uncoupling, generating superoxide rather than NO. This redox imbalance promotes oxidative stress and induces non-apoptotic cell death [15]. Similarly, excessive nNOS activation in GPR55 activation models induces NO-dependent apoptosis, further reinforcing its tumor-suppressing potential [16]. Conversely, in ovarian cancer, nNOS enhances glycolysis by S-nitrosylating phosphofructokinase (PFK), promoting tumor proliferation and metastasis [17]. This functional inconsistency highlights the context-dependent nature of nNOS, where its role varies significantly across different tissue environments. Currently, the specific mechanisms of nNOS in HB, a malignancy characterized by abnormal proliferation, remain unclear. Given the urgent need to understand the molecular drivers of HB progression, elucidating the role of nNOS in this context is essential for advancing targeted therapeutic strategies.

To investigate the role of nNOS in HB cell proliferation, we performed an integrated analysis of the nNOS-regulated transcriptome and proteome, complemented by in vitro and in vivo experiments. Our findings demonstrated that overexpression of nNOS in HB cells reduces KRAS expression, inhibits MAPK pathway activation, and consequently suppresses HB cell proliferation. This evidence underscores the potential of nNOS as a therapeutic target in HB and highlights the need for further exploration of its molecular mechanisms.

nNOS generates NO, which covalently binds to cysteine thiols, inducing S-nitrosylation of target proteins [18,19]. This modification is crucial for regulating protein function in both physiological and pathological conditions [[20], [21], [22], [23]]. Our previous research demonstrates that nNOS can suppress autophagy in nasopharyngeal carcinoma cells by S-nitrosylating PTEN and activating the Akt/mTOR pathway [24]. Additionally, nNOS promotes S-nitrosylation of GAPDH, inducing its nuclear translocation and triggering apoptosis in colorectal cancer cells [25]. Furthermore, nNOS-induced S-nitrosylation of PFK enhances glycolysis in ovarian cancer cells [26]. These findings underscore the key role of nNOS in regulating cancer cell autophagy, apoptosis, and glycolysis through S-nitrosylation. However, the impact of nNOS-induced S-nitrosylation on HB cell proliferation and the underlying mechanisms remain an open question that warrants further investigation.

In our mechanistic study, we found that nNOS modulates treacle ribosome biogenesis factor 1 (TCOF1) through S-nitrosylation. TCOF1, a key nucleolar protein that regulates ribosomal DNA transcription and plays a vital role in growth and development [[27], [28], [29]], interacts with KRAS to stabilize it. However, S-nitrosylation of TCOF1 attenuates this interaction, leading to reduced KRAS protein expression and subsequent suppression of HB cell proliferation. This discovery highlights a novel regulatory mechanism involving nNOS in HB progression.

## 2 Materials and methods

### 2.1 Antibodies and reagents

Antibodies and reagents are listed in Tables S1 and S2, respectively.

### 2.2 Cell line and culture

Human HB (Huh6, HepG2) and human embryonic kidney (HEK-293T) cells were obtained from the Cell Bank of the Chinese Academy of Sciences (Shanghai, China). Cells were maintained in RPMI-1640 (#12633020, Gibco) or DMEM (#12491023, Gibco) supplemented with 10 % fetal bovine serum (FBS) (#10099141, Gibco) and 1 % penicillin–streptomycin (#15140122, Gibco) at 37 °C in a humidified 5 % CO_2_ atmosphere.

### 2.3 Collection and processing of clinical specimens

Tumor and matched normal liver tissue (>2 cm from margin) were obtained from pediatric HB patients, fixed in formalin, and paraffin-embedded. The protocol was approved by the IRB of Guangzhou Women and Children's Medical Center (No. G2023-396) and adhered to the Declaration of Helsinki (2013); informed consent was provided by legal guardians.

### 2.4 Animals

Male BALB/c nude mice (6–8 weeks, 18–22 g; Guangzhou Rige Biotechnology) were injected subcutaneously in the right flank with 1 × 10^6^ Huh6 cells in 100 μL PBS. Tumor length and width were measured every 3 days with calipers; volume = (length × width^2^)/2. Mice were euthanized at ∼100 mm^3^. Animals were housed SPF with autoclaved food and water. Procedures were approved by Guangzhou Bojin IACUC (protocol 2022-KY-1402-001) and followed NIH guidelines.

### 2.5 S- Nitrosoprotein detection by biotin-switch assay

Cells were lysed in ice-cold HEN buffer (25 mM HEPES, pH 7.4; 50 mM NaCl; 0.1 mM EDTA; 1 % NP-40; protease inhibitor cocktail). Lysates were clarified at 12 000×*g*, 15 min, 4 °C, and protein concentration was determined by BCA. Equal amounts (2 mg) were processed for each condition. Free thiols were blocked with 15 mM S-methyl methanethiosulfonate (MMTS; Thermo Fisher, 23011) in 2.5 % SDS (1:10 dilution) at 50 °C for 15 min. Proteins were precipitated with cold acetone (−20 °C, 30 min) and pelleted at 8000×*g*, 10 min, 4 °C. The pellet was resuspended in HEN buffer containing 1 % SDS. Samples were incubated with or without 20 mM sodium ascorbate for 1 h at room temperature in the dark, followed by labeling with EZ-Link HPDP-biotin (2.5 mg/mL; Thermo Fisher, 21341) for 1 h. Ten percent of the labeled lysate was reserved as input; the remainder was incubated with streptavidin agarose resin (Yeasen, 20512ES08) at 4 °C for 2 h with gentle rotation. Resin was washed three times with PBS containing 0.1 M NaOH and 0.1 % Tween-20, and bound proteins were eluted for SDS-PAGE and Western blot analysis.

### 2.6 Immunohistochemistry (IHC) staining and scoring criteria

4 μm FFPE sections were baked (60 °C, 2 h), dewaxed, rehydrated, and subjected to antigen retrieval (0.01 M citrate, pH 6.0, 95–98 °C, 20 min). Endogenous peroxidase was quenched (3 % H_2_O_2_, 10 min, RT) and sections blocked (10 % goat serum, 1 h). After overnight incubation with *anti*-nNOS (4 °C) and HRP-secondary (30 min, RT), signals were developed with DAB (5 min) and counterstained with hematoxylin. Two blinded pathologists scored staining: % positive cells (1, 0–25 %; 2, 26–50 %; 3, 51–75 %; 4, 76–100 %) × intensity (0–3) = H-score (0–12); 0–6 = low, 7–12 = high.

### 2.7 qRT-PCR

Total RNA was isolated with RNA Isolater Total RNA Extraction Reagent (Vazyme). First-strand cDNA was synthesized using the All-in-One RT SuperMix Perfect RT-PCR Kit (Vazyme). Gene expression was quantified on an Applied Biosystems QuantStudio 6 Flex system with SYBR Green Master Mix (Vazyme). Results were normalized to ACTB. Primer sequences are listed in Table S1.

### 2.8 EdU incorporation assay

Huh6 cells were seeded in 6-well plates. After 24 h, medium was replaced with EdU working solution (EdU Imaging Kit, Cy5; APExBIO #K1076) for the indicated time. Cells were fixed (4 % PFA), permeabilized (0.1 % Triton X-100), and subjected to the Click-iT reaction (30 min, RT, dark). Nuclei were stained with Hoechst 33342 (10 min, RT, dark), washed, and imaged by fluorescence microscopy. EdU-positive cells were counted and expressed as a fraction of total cells.

### 2.9 Western blot

Cells were lysed on ice in RIPA buffer with 1 % protease inhibitor (FudeBIO, Hangzhou, China). After BCA quantification, 20–30 μg protein per lane was resolved on 10 % SDS-PAGE and transferred overnight to PVDF at 4 °C. Membranes were blocked (5 % milk in TBST, 1 h, RT), incubated with primary antibodies overnight at 4 °C, washed three times with TBST, and probed with HRP-conjugated secondary antibodies (1 h, RT). Signals were developed with FDbio-Dura ECL and quantified in ImageJ; data are from three independent experiments.

### 2.10 Co-immunoprecipitation (Co-IP)

Cells were lysed in ice-cold buffer containing protease inhibitors and clarified (12 000×*g*, 10 min, 4 °C). One milligram of supernatant was incubated overnight at 4 °C with 1 μg primary antibody plus 1 μg rabbit IgG, followed by 2 h with 50 μL Protein A/G magnetic beads (Pierce kit 88804). Beads were washed three times with PBST and eluted in 2 × Laemmli buffer for Western blot.

### 2.11 Plasmid construction, lentivirus production, and stable cell line generation

Two 21-nt shRNAs against TCOF1 (5-GACGCTTCATATAGATGTGTA-3′and 5′-GAGTCATCAGACAGCAGTGAT-3) were cloned into pLKO.1-TRC (TSINGKE). nNOS cDNA was inserted into GV492 (Genechem). WT and C38S/C644S TCOF1 variants were sub-cloned into pcDNA3.1-3 × FLAG and pPB-EF1A-mCherry-Hygro (AISEN Gene). iNOS, GSNOR and KRAS were cloned into pcDNA3.1-HA (TSINGKE). Empty vectors and scrambled shRNA served as controls.

For lentivirus production, HEK293T cells were co-transfected with target plasmid plus psPAX2 and pMD2.G using Lipofectamine 3000 (Invitrogen). Supernatants collected at 48 and 72 h were pooled, concentrated by ultracentrifugation, and used to infect target cells in the presence of 8 μg/mL Polybrene for 12 h. Stable lines were selected with 2 μg/mL puromycin or 800 μg/mL hygromycin.

### 2.12 Cell proliferation assays

Cells were plated at 3 × 10^3^ cells per well in 96-well plates and cultured under standard conditions. At 24, 48, and 72 h, 10 μL CCK-8 reagent (GLPBIO, USA) was added to each well and incubated at 37 °C for 2 h. Absorbance at 450 nm was recorded on a microplate reader. All measurements were performed in triplicate.

### 2.13 Immunofluorescence (IF) and confocal microscopy

Cells on glass-bottom dishes were fixed (4 % PFA, 15 min, RT), permeabilized (0.5 % Triton X-100, 5 min, RT) and blocked (5 % BSA in PBS, 1 h). Primary antibodies were applied overnight at 4 °C; after three 10-min PBS washes, fluorophore-conjugated secondary antibodies were added (1 h, RT, dark). Nuclei were stained with DAPI (5 min), washed, and imaged on a confocal microscope. Fluorescence intensity and colocalization were quantified in ImageJ after background subtraction.

### 2.14 Caspase-3 activity assay

Caspase-3 activity was measured with the Caspase-3 Activity Assay Kit (Beyotime, Shanghai, China). Cells were lysed in ice-cold buffer containing 1 mM DTT, clarified (16 000×*g*, 15 min, 4 °C), and protein adjusted to 20–30 μg per reaction. Reactions were started with 2 mM Ac-DEVD-pNA and incubated at 37 °C for 90 min pNA release was measured at 405 nm against a 0–200 μM standard (R^2^ > 0.99) and expressed as nmol pNA/(h·mg) protein. Assays were run in technical triplicate.

### 2.15 NO detection assays

Total NO metabolites: Cell lysates were cleared (12 000×*g*, 10 min, 4 °C) after precipitation with 1 % ZnSO_4_. Supernatant (50 μL) was reacted with Griess-VCl_3_ reagent (30 min, 37 °C, dark) and absorbance was read at 540 nm. Concentrations (nmol NO mg per protein) were calculated against a NaNO_2_ standard curve.

Endogenous NO imaging: Cells were loaded with BBoxiProbe® O38 (1:1000 in HBSS, 30 min, 37 °C, dark), washed twice with PBS, and imaged by confocal microscopy (excitation/emission wavelengths: 510/610 nm). Fluorescence intensity was quantified in ImageJ after background subtraction.

### 2.16 Superoxide anion detection assay

Reactive Oxygen Species Assay Kit for Superoxide Anion with Dihydroethidium (DHE, Beyotime, S0063) was used to detect intracellular superoxide anion through the specific oxidation of DHE to generate red fluorescent products. After washing with PBS, cells were incubated with 5 μM DHE working solution at 37 °C for 30 min in the dark. The positive control group was pre-treated with 5 μM Rosup II for 60 min to induce oxidative stress. Following staining, cells were washed with PBS and observed under a fluorescence microscope (excitation/emission wavelengths: 535/610 nm) to assess fluorescence intensity.

### 2.17 Quantitative S-Nitrosoproteomics using IodoTMT labeling and LC-MS/MS

Huh6 cells ± nNOS overexpression were lysed (1 % SDS, protease inhibitors, 25 mM IAM) by sonication. Free thiols were blocked with 25 mM IAM (1 h, RT, dark); debris was removed (12 000×*g*, 10 min, 4 °C). After BCA normalization, 1 mg protein per sample was processed with the iodoTMT 6-plex kit (Thermo 90102): reduction with 10 mM ascorbate, labeling (37 °C, 1 h, dark), quenching with 20 mM DTT, acetone precipitation, and tryptic digestion (1:50, w/w, 200 mM TEAB, 37 °C, 16 h). Peptides were reduced (5 mM DTT, 56 °C, 30 min), alkylated (11 mM IAM, 15 min, RT), enriched on *anti*-TMT resin (4 °C, 16 h), washed, and eluted (50 % ACN, 0.4 % TFA). After C18 ZipTip desalting, samples were analyzed on an EASY-nLC 1200 coupled to a Q Exactive HF-X (Thermo). Full MS (350–1500 *m/z*, 120 000 resolution) was followed by HCD-MS/MS (NCE 28 %, top 20, 15 000 resolution, 30 s dynamic exclusion). Data were searched with MaxQuant v1.5.2.8 against the human UniProt database (1 % FDR).

### 2.18 Quantitative proteomic analysis

Sample preparation, TMT labeling (10-plex), LC–MS/MS and data processing were performed by Jingjie Biotechnology (Hangzhou, China). Peptides were analyzed on an EASY-nLC 1200 coupled to a Q Exactive HF-X (Thermo). MaxQuant v2.1.0 was used to search the UniProt human proteome (release 2023_01, 1 % FDR).

### 2.19 Molecular docking

AlphaFold2-derived structures of TCOF1 (UniProt ID: Q13428) and KRAS (UniProt ID: P01116) were docked using ClusPro 2.0 with default settings. The lowest-energy pose among the top 30 ranked by PIPER energy was selected as the optimal complex.

### 2.20 Transcriptome sequencing (RNA-seq)

Total RNA was extracted with the MJzol kit (Majorivd) and cleaned with RNAClean XP beads (Beckman Coulter) plus DNase I (QIAGEN). RNA integrity (RIN ≥7), concentration (Qubit 2.0) and purity (NanoDrop; A260/280 1.8–2.1, A260/230 ≥ 2.0) were verified. Poly-A mRNA was fragmented, reverse-transcribed, end-repaired, A-tailed, and ligated to adapters. Libraries (250–350 bp) were quantified on TapeStation and sequenced 2 × 150 bp on Illumina NovaSeq 6000; raw reads were processed with Illumina's standard pipeline.

### 2.21 Statistical analysis

Data are presented as mean ± standard deviation (SD) of at least three independent experiments. Statistical tests (specified in figure legends) were performed with GraphPad Prism v9 (GraphPad Software, San Diego, CA). Significance: ∗P < 0.05, ∗∗P < 0.01, ∗∗∗P < 0.001; ns, not significant.

## 3 Results

### 3.1 nNOS Downregulation Negatively Correlates with HB progression

**Fig. 1:**
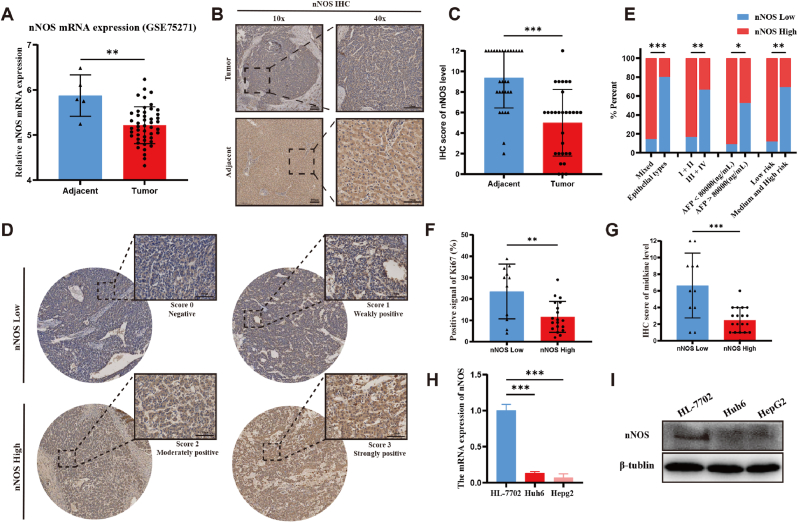
nNOS Downregulation Negatively Correlates with HB Progression. (A) nNOS mRNA expression levels in HB tissues versus normal liver tissues, analyzed via the GSE75271 dataset from the GEO database. (B) Representative IHC staining images of nNOS expression in HB tissues and adjacent normal liver tissues (scale bar, 50 μm). (C) Quantification of nNOS expression in HB and adjacent normal tissues by IHC scoring (n = 30). (D) Classification of HB patients into low-nNOS and high-nNOS groups based on IHC staining scores. Representative images of low and high nNOS expression groups are shown (Score 0: Negative; Score 1: Weakly positive; Score 2: Moderately positive; Score 3: Strongly positive). scale bar, 50 μm (E) Correlation analysis of nNOS expression with clinical characteristics, including PRETEXT staging, AFP levels, histological subtypes, and risk stratification. (F, G) IHC analysis of proliferation markers Ki67 and midkine in tumor tissues with low versus high nNOS expression levels. (H) qRT-PCR determination of nNOS mRNA expression levels in HL-7702, Huh6 and HepG2. (I) Western blot analysis of nNOS protein expression levels in HL-7702, Huh6 and HepG2. β-Tubulin served as a loading control. The results are shown as the mean ± SD obtained from three independent experiments. Statistical analysis was performed using independent or paired t-tests and Chi-Square test. Statistical significance was defined as ∗P < 0.05, ∗∗P < 0.01, ∗∗∗P < 0.001. ns, not significant.

To investigate the role of nNOS in the development of HB, we analyzed nNOS mRNA expression levels using the GSE75271 dataset (n = 50 HB vs.5 normal liver tissues) from the Gene Expression Omnibus (GEO). Our findings revealed a significant downregulation of nNOS mRNA expression in HB compared to normal liver tissue (Fig. 1A). This downregulation was further validated through IHC analysis of tumor and adjacent normal liver tissues from 30 HB patients (Fig. 1B–C), confirming significantly lower nNOS expression in HB tissues. Based on nNOS staining scores, patient samples were divided into low-expression (N = 11) and high-expression (N = 19) groups (Fig. 1D). Correlation analysis revealed significant associations between nNOS expression and clinical features such as PRETEXT stage, AFP levels, HB histological types, and risk stratification (Table 1 and Fig. 1E). Moreover, IHC analysis of tumor tissues showed a negative correlation between low nNOS expression and the expression of proliferation markers Ki67 and midkine (Fig. 1F–G). Using qRT-PCR and Western blot, we further assessed nNOS expression in normal liver cells (HL-7702) and HB cells (Huh6 and HepG2). The results indicated lower nNOS expression in HB cell lines compared to normal liver cells (Fig. 1H–I). Collectively, these findings support the notion that nNOS may function as a suppressor in HB development, highlighting its potential as a therapeutic target for HB treatment.

**Table 1:** Correlation of nNOS Expression with Clinicopathological Features in HB Patients.

Characteristic	Number of patients	nNOS protein expression		P-value
	Total	Low	High	
**Gender**				0.2663
Male	18	5	13	
Female	12	6	6	
**Age**				0.4189
<12 months	9	2	7	
≥12 months	21	9	12	
**Tumor Size**				0.6722
≥500 cm^3^	22	9	13	
<500 cm^3^	8	2	6	
**Histological types of HB**				**0.001**
Mixed	20	3	17	
Epithelial types	10	8	2	
**PRETEXT**				**0.0086**
I + II	18	3	15	
III + IV	12	8	4	
**Metastasis**				0.1563
–	24	7	17	
+	6	4	2	
**Risk stratification**				**0.0021**
Low	17	2	15	
Medium and High	13	9	4	
**AFP (ng/mL)**				**0.0231**
<80000	11	1	10	
>80000	19	10	9	
**CD34**				0.1322
+	19	6	13	
–	11	7	4	

### 3.2 nNOS Overexpression Suppresses HB Cell Proliferation In Vivo and In Vitro

**Fig. 2:**
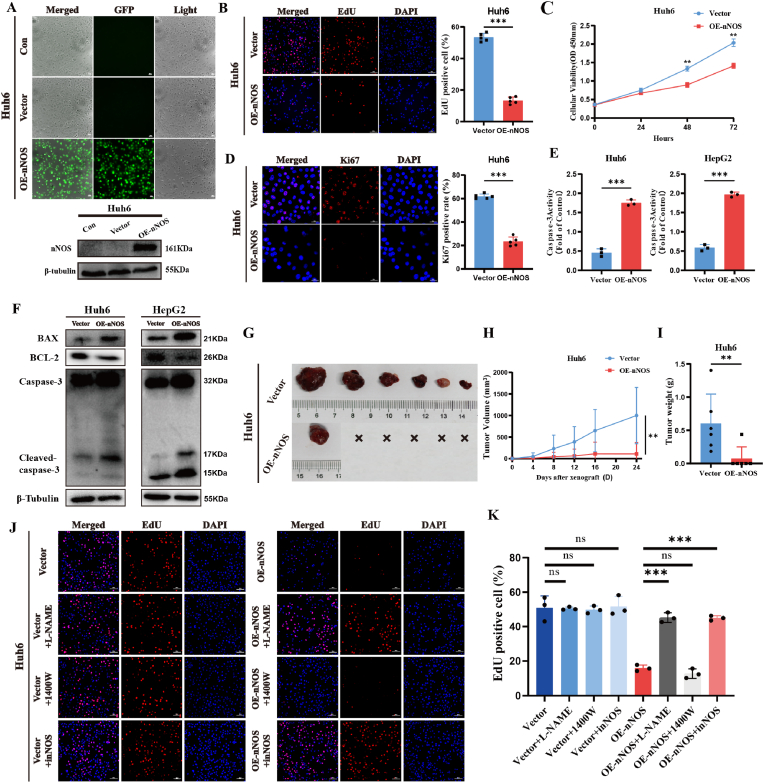
nNOS Overexpression Suppresses HB Cell Proliferation In Vivo and In Vitro. (A) Confirmation of nNOS protein expression in Huh6 cells via Western blot and IF microscopy (GFP, green) (scale bar, 100 μm). Cells were transfected with either an empty vector (vector group) or an nNOS lentiviral overexpression vector (OE-nNOS group). EdU staining (B) (scale bar, 100 μm), CCK-8 assay (C), and Ki67 IF (D) (scale bar, 50 μm) were utilized to evaluate the proliferative capacity of Huh6 cells with nNOS overexpression compared to control cells. Quantification of EdU- and Ki67-positive cells is shown on the right. (E) Caspase-3 activity assay in Huh6 and HepG2 cells transfected with either vector or OE-nNOS. (F) Western blot analysis of BAX, BCL-2, Caspase-3, and Cleaved caspase-3 in Huh6 and HepG2 cells transfected with vector or OE-nNOS.β-Tubulin served as a loading control. (G) Representative images of xenograft tumors in nude mice subcutaneously injected with Huh6 cells stably transfected with nNOS or empty vector. (H–I) Tumor growth curve illustrating the effect of nNOS overexpression on tumor volume in the xenograft model, and tumor weight at the experiment's conclusion. (J) EdU staining was performed to verify the proliferation of vector and OE-nNOS Huh6 cell groups, each treated with 1 μM l-NAME, 2 μM 1400W and 2 μM inNOS (scale bar, 50 μm). (K) Quantification of EdU Staining Results. Data are presented as mean ± SD from three independent experiments. Statistical analysis was performed using independent t-tests and two-way ANOVA. Significance levels are indicated as ∗P < 0.05, ∗∗P < 0.01, ∗∗∗P < 0.001; ns, not significant.

Based on nNOS expression levels in HB cell lines, we chose Huh6 and HepG2 cells for stable nNOS overexpression and examined its impact on cell proliferation both in vitro and in vivo. Fluorescence Microscopy Imaging and Western blot analyses confirmed the overexpression efficiency (Fig. 2A, Fig. S1A). EdU and CCK8 assays demonstrated that nNOS overexpression inhibits the proliferation of Huh6 and HepG2 cells (Fig. 2B–C, Fig. S1B–C). Consistently, IF results revealed that Ki67 fluorescence intensity was markedly lower in nNOS-overexpressing cells than in control cells (Fig. 2D, Fig. S1D). To investigate the relationship between nNOS and cell apoptosis, we measured the activity of Caspase-3, a key enzyme in apoptosis, and found that nNOS significantly inhibits Caspase-3 activity (Fig. 2E). Subsequent Western blot results also confirmed that nNOS significantly inhibits the expression of the anti-apoptotic protein BCL-2, while promoting the expression of the pro-apoptotic protein BAX and Cleaved caspase-3 (Fig. 2F). These in vitro experiments establish that nNOS inhibits HB cell proliferation and promotes apoptosis. We next established a tumor xenograft model to further evaluate the effect of nNOS overexpression on tumor growth in vivo. Analysis of tumor growth curves and final tumor weight demonstrated that nNOS overexpression significantly inhibits tumor growth in Huh6 cells (Fig. 2G–I), proving that nNOS inhibits tumor proliferation in vivo. Studies have linked high inducible nitric oxide synthase (iNOS) expression to poor prognosis and metastasis in HB [30]. To test whether the previously observed anti-proliferative effect is restricted to nNOS rather than a generic NOS response, we engineered Huh6 cells to stably overexpress iNOS. Western blot confirmed efficient transfection (Fig. S2A); however, EdU and CCK-8 assays demonstrated that iNOS overexpression accelerated proliferation (Fig. S2B–D), mirroring the nNOS-mediated inhibition in reverse. Isoform specificity was interrogated pharmacologically. Cells were treated in parallel with the pan-NOS blocker l-NAME, the iNOS-selective antagonist 1400W, or the nNOS-selective inhibitor nNOS-Inhibitor I (abbreviated as inNOS). EdU incorporation indicated that nNOS-driven growth arrest was partially reversed by l-NAME or inNOS, whereas 1400 W was without effect (Fig. 2J–K, Fig. S1E). In contrast, iNOS-induced proliferation was curtailed by l-NAME and 1400W but remained impervious to inNOS (Fig. S2C–D). Collectively, these results establish nNOS as the principal suppressor of HB cell proliferation both in vitro and in vivo.

### 3.3 Transcriptomic and Proteomic Analysis Reveals nNOS suppresses HB cell proliferation via MAPK Pathway Inhibition

**Fig. 3:**
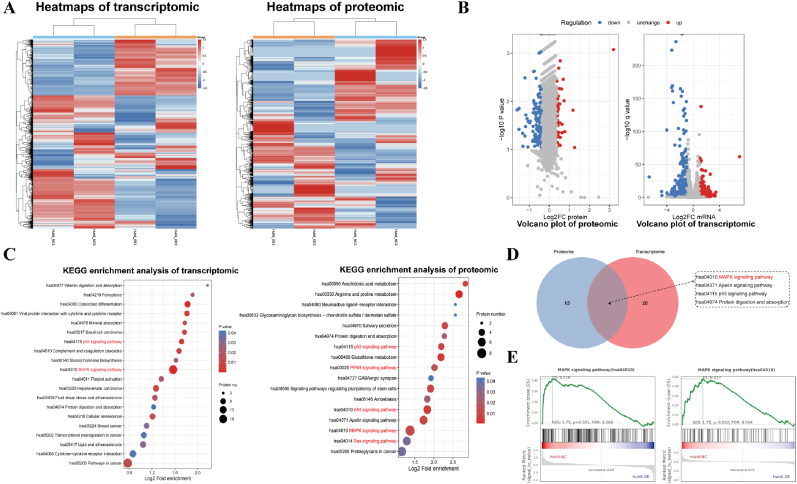
Transcriptomic and Proteomic Analysis Reveals nNOS Suppresses HB Cell Proliferation via MAPK Pathway Inhibition. (A) Heatmaps depicting DEGs and DEPs in Huh6 cells overexpressing nNOS versus control cells, with a color gradient from blue (low expression) to red (high expression). (B) Volcano plots showing DEGs and DEPs, where red dots indicate upregulated genes/proteins and blue dots indicate downregulated genes/proteins. (C) KEGG pathway enrichment analysis of DEGs (left panel) and DEPs (right panel). (D) A Venn diagram illustrating the overlap of significantly enriched KEGG pathways from both transcriptomic and proteomic analyses. (E) GSEA of RNA-seq and proteomics data independently demonstrated suppression of MAPK signaling pathway activity in nNOS-overexpressing Huh6 cells. Normalized enrichment scores (NES) and p-values are provided.

To explore the potential role of nNOS in inhibiting HB cell proliferation, we conducted transcriptomic and TMT-based quantitative proteomic analyses on nNOS-overexpressing Huh6 cells and control cells [31]. RNA sequencing and proteomic data quality was confirmed via PCA (Fig. S3A–B). In the transcriptomic analysis, compared to the control cells, there were 183 genes significantly upregulated and 312 genes significantly downregulated in nNOS-overexpressing Huh6 cells. The criteria for screening differentially expressed genes (DEGs) were a fold change >2 and a coefficient of variation (CV) > 0.2, which are presented in heatmaps (Fig. 3A) and volcano plots (Fig. 3B). Similarly, in the proteomic analysis, there were 37 proteins significantly upregulated and 150 proteins significantly downregulated in nNOS-overexpressing Huh6 cells compared to the control cells. The criteria for screening differentially expressed proteins (DEPs) were a fold change >2 and a coefficient of variation (CV) > 0.2, and these results are also presented in heatmaps (Fig. 3A) and volcano plots (Fig. 3B). GO and KEGG enrichment analyses were performed on both datasets. KEGG analysis revealed altered expression in pathways related to HB cell proliferation, including MAPK, Ras, p53, and Wnt pathways (Fig. 3C). GO analysis showed significant enrichment of cell proliferation-related biological processes (Fig. S3C–D). Intersection analysis found 4 overlapping pathways in KEGG results, with MAPK, Apelin, and p53 pathways being closely related to cell proliferation (Fig. 3D). GSEA further demonstrated downregulated MAPK pathway activity in nNOS-overexpressing cells at both transcriptomic and proteomic levels (Fig. 3E). Given the MAPK pathway's key role in cell proliferation, we hypothesize that nNOS inhibits HB cell proliferation by suppressing MAPK pathway activity.

### 3.4 nNOS Overexpression Suppresses KRAS to regulate MAPK signaling

**Fig. 4:**
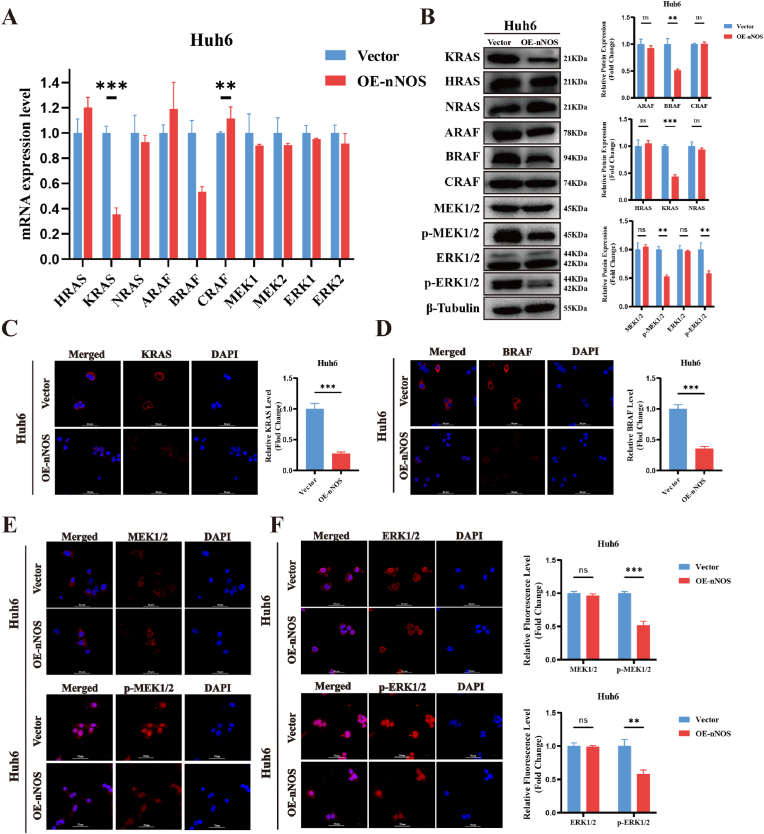
nNOS Overexpression Suppresses KRAS to Regulate MAPK Signaling. (A) Relative mRNA expression levels of MAPK pathway-related genes, including HRAS, KRAS, NRAS, ARAF, BRAF, CRAF, MEK1/2, and ERK1/2, in OE-nNOS Huh6 cells or vector control. (B) Western blot analysis of the protein expression levels of MAPK pathway components, including HRAS, KRAS, NRAS, ARAF, BRAF, CRAF, MEK1/2, p-MEK1/2, ERK1/2 and p-ERK1/2, in the same experimental groups. β-Tubulin was used as a loading control. Representative blots (left) and corresponding quantified data (right) are shown. (C–F) IF staining of KRAS (C), BRAF (D), MEK1/2 and p-MEK1/2 (E), ERK1/2 and p-ERK1/2 (F) in the same experimental groups. Representative images of those indicators with DAPI-stained nuclei (blue) are shown, along with quantified fluorescence intensity (right panels). The results are shown as the mean ± SD obtained from three independent experiments. Statistical analysis was performed using Student's *t*-test. Statistical significance was defined as ∗P < 0.05, ∗∗P < 0.01, ∗∗∗P < 0.001. ns, not significant. Scale bar, 50 μm.

Transcriptomic and proteomic sequencing results indicated that nNOS overexpression inhibits HB cell proliferation by targeting the MAPK signaling pathway. To validate this hypothesis, we focused on the MAPK pathway as the key target and examined the expression of MAPK-related proteins upon nNOS overexpression to clarify its regulatory role. qRT-PCR and Western blot analyses revealed that nNOS overexpression significantly reduced phosphorylation levels of ERK1/2 and MEK1/2 in Huh6 and HepG2 cells. This was accompanied by marked downregulation of upstream BRAF and KRAS expression. In contrast, other RAS family members (HRAS, NRAS) and RAF family members (ARAF, CRAF) showed no significant changes (Fig. 4A–B, Fig. S4A–B). Subsequent IF assays further confirmed that nNOS overexpression substantially suppressed BRAF, KRAS, p-ERK1/2, and p-MEK1/2 expression (Fig. 4C–F, Fig. S4C–F).

### 3.5 Global Analysis of Protein S-Nitrosylation Mediated by nNOS in Huh6 cells

nNOS overexpression can alter subcellular localization and catalytic coupling in cardiomyocytes, thereby modifying NO-dependent cardiac regulation [32]. Under pathological conditions, uncoupled nNOS shifts from NO generation to superoxide (O_2_^−^) production [33]. To exclude aberrant localization or enzyme dysfunction in our model, we compared nNOS distribution in normal hepatocytes (HL-7702), Huh6, and Huh6 OE-nNOS. IF revealed perinuclear and cytoplasmic nNOS in HL-7702 cells; OE-nNOS Huh6 cells displayed an identical pattern, confirming that overexpression does not perturb localization (Fig. S5A). Endogenous nNOS was barely detectable in wild-type Huh6 cells. Intracellular NO levels were quantified using the NO-selective fluorescent probe BBoxiProbe® O38 and Griess-based total nitrite/nitrate assays. Both methods confirmed a significant increase in NO levels upon nNOS overexpression (Fig. S5B–C). To assess superoxide generation, cells were incubated with DHE. Relative to the positive control (1 μM Rosup II), DHE fluorescence remained low in OE-nNOS Huh6 cells, indicating that nNOS overexpression does not elevate endogenous superoxide levels (Fig. S5D).

**Fig. 5:**
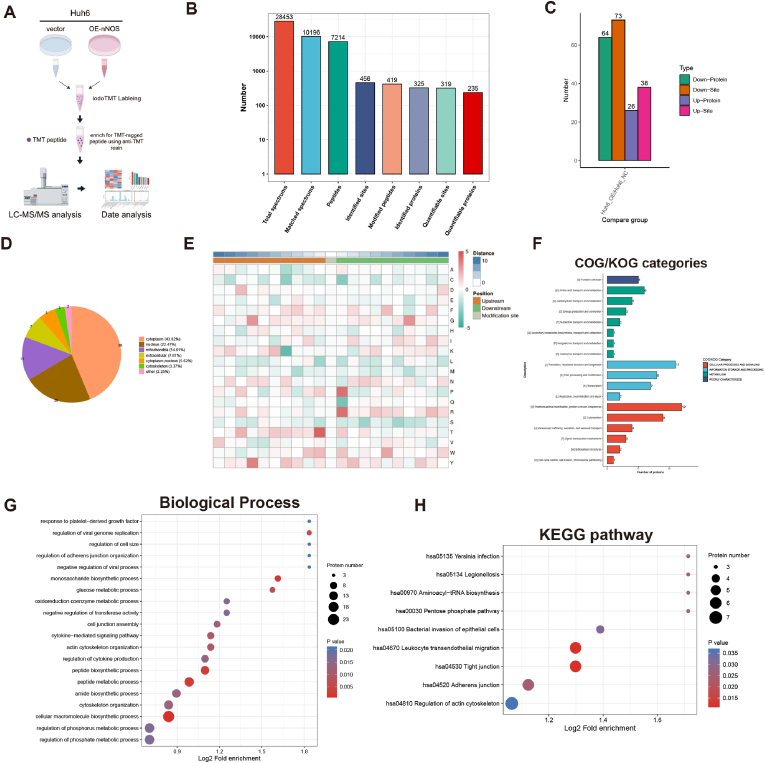
Global Analysis of Protein S-Nitrosylation Mediated by nNOS in Huh6 cells. (A) S-nitrosylation proteomics detection workflow diagram. (B) Analysis of mass spectrometry data to determine the number of identified S-nitrosylation sites, peptides, and proteins. (C) Statistical analysis of differentially S-nitrosylated proteins and modification sites in Huh6 cells overexpressing nNOS compared to control cells. (D) Subcellular distribution of S-nitrosylated proteins. (E) Heatmap illustrating amino acid distribution, showing enrichment (red) and depletion (green) around S-nitrosylation sites. (F) COG/KOG enrichment analysis of S-nitrosylated proteins. (G) GO biological process enrichment analysis of S-nitrosylated proteins. (H) KEGG pathway enrichment analysis revealing various pathways potentially involved in S-nitrosylation modifications.

Protein S-nitrosylation, a key post-translational modification, precisely regulates protein function and activity by modulating the redox state of cysteine thiols [[34], [35], [36]]. Elevation of intracellular NO is the principal trigger for protein S-nitrosylation. To investigate the impact of nNOS on the global S-nitrosylation landscape in tumor cells, we employed TMT labeling, immunoenrichment, and LC-MS/MS to quantify the S-nitrosylation of proteins in Huh6 cells overexpressing nNOS [37] (Fig. 5A). The relative standard deviation (RSD) of replicates within each group confirmed the good reproducibility of the S-nitrosylated proteomics data (Fig. S6A). Principal component analysis (PCA) revealed distinct clustering of control and nNOS-overexpressing groups (Fig. S6B). Product analysis showed that most peptide fragments carried 2–4 charges and had lengths of 7–20 amino acids, meeting proteomic quality standards (Fig. S6C). In Huh6 cells, we identified 456 SNO sites in 325 proteins and precisely quantified 319 SNO sites in 235 proteins (Fig. 5B). Upon nNOS overexpression, 111 SNO sites in 90 proteins showed significant changes (Fig. 5C), as visualized by heat maps (Fig. S6D). Bioinformatics analysis revealed that nearly half (43.08 %) of the SNO-modified proteins were located in the cytoplasm (Fig. 5D). Analysis of the amino acid sequences surrounding S-nitrosylation sites revealed significant enrichment of specific amino acids at particular positions: threonine (Thr) at position −1 and proline/arginine (Pro/Arg) at position +1 (Fig. 5E). This suggests a preference for certain amino acid microenvironments in S-nitrosylation. Functional enrichment analysis uncovered a multi-layered regulatory network of S-nitrosylation. KOG classification showed that modified proteins were involved in cell cycle control, cell division, and signal transduction mechanisms (Fig. 5F). GO enrichment analysis revealed that S-nitrosylation potentially governs proliferation by modulating the platelet-derived growth factor signaling pathway (Fig. 5G, Fig. S6E–F). KEGG analysis further demonstrated that S-nitrosylated proteins are significantly enriched in aminoacyl-tRNA biosynthesis and the pentose phosphate pathway—metabolic routes that provide essential precursors and NADPH for anabolic growth and division—thereby indirectly fueling tumor cell proliferation (Fig. 5H).

### 3.6 nNOS inhibits HB cell proliferation via TCOF1 S-nitrosylation

**Fig. 6:**
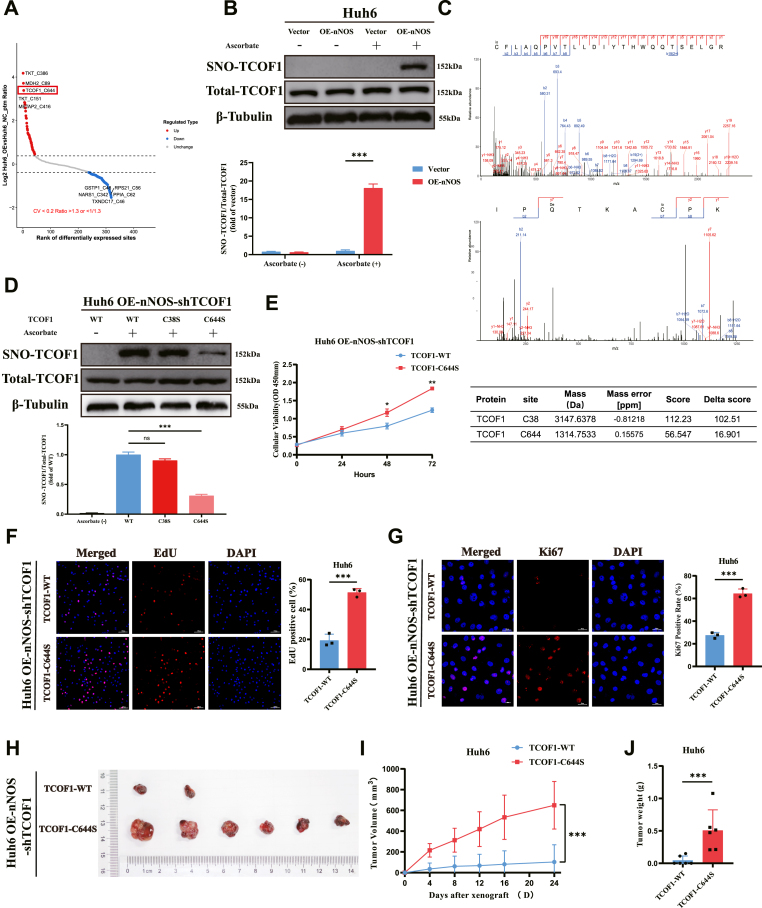
nNOS Inhibits HB Cell Proliferation via TCOF1 S-Nitrosylation. (A) Volcano plot showing differentially S-nitrosylated proteins in Huh6 cells overexpressing nNOS, with emphasis on significant upregulation of TCOF1 S-nitrosylation at C644 (highlighted by a red box). (B) Biotin-switch assay for SNO-TCOF1 in OE-nNOS and vector Huh6 lysates, followed by total-TCOF1 immunoblot. A reducing-agent-free lane served as the negative control; β-tubulin was the loading control. Quantification (densitometry) is shown on the right. (C) Mass spectrometry identified S-nitrosylation of TCOF1 at Cys38 and Cys644. S-nitrosation modified peptides is shown, including calculated monoisotopic mass (mass), mass errors measured in parts per million (ppm), score and Delat score. (D) Detection of SNO-TCOF1 by biotin switch assay with Western blot verification of total TCOF1 levels in nNOS-overexpressing Huh6 cells subjected to TCOF1 knockout followed by reconstitution with either wild-type TCOF1 (TCOF1-WT) or cysteine-mutant TCOF1 (TCOF1-C38S/C644S). A lane without ascorbate served as the negative control; β-tubulin was the loading control. Densitometry is shown below. (E–G) CCK-8 assay (E), EdU staining (F) (scale bar, 100 μm) and Ki67 IF (G) (scale bar, 50 μm) were used to assess proliferation of nNOS-overexpressing, TCOF1-knockout Huh6 cells reconstituted with TCOF1-WT or TCOF1-C644S. Quantification of EdU- and Ki67-positive cells is shown on the right. (H) Xenograft images from nude mice injected subcutaneously with TCOF1-knockout, nNOS-overexpressing Huh6 cells stably reconstituted with TCOF1-WT or TCOF1-C644S. (I–J) Tumor growth curves and tumor weight indicated that the TCOF1-C644S mutation partially counteracted the inhibitory effect of nNOS on cell proliferation. Data indicated as mean ± SD (n = 6 mice per group). Data are mean ± SD of three independent experiments. Statistics: Student's t-test and two-way ANOVA; ∗P < 0.05, ∗∗P < 0.01, ∗∗∗P < 0.001; ns, not significant.

Volcano plot analysis revealed significantly increased TCOF1 S-nitrosylation levels under nNOS regulation, with the modification site identified as C644 (Fig. 6A). Literature shows TCOF1 affects KRAS activation in liver cancer [38], prompting our speculation that nNOS may influence KRAS expression by modulating TCOF1 S-nitrosylation. To verify the S-nitrosylation results of TCOF1, we used the biotin switch assay to detect its S-nitrosylation level in Huh6 cells. nNOS overexpression increased this level, without affecting TCOF1 protein expression (Fig. 6B), consistent with proteomic data. Co-IP confirmed the absence of nNOS binding to either KRAS or TCOF1 (Fig. S7A). Furthermore, ectopic iNOS expression in Huh6 cells did not elicit TCOF1 S-nitrosylation (Fig. S7B), indicating that this modification is nNOS-specific and dependent on elevated intracellular NO. To determine the potential S-nitrosation sites of TCOF1, we analyzed quantitative proteomics data and identified two cysteine residues (Cys38 and Cys644) as potential S-nitrosation sites (Fig. 6C). To determine which of these sites plays a role in nNOS-mediated proliferation of HB cells, we constructed TCOF1 expression vectors with individual cysteine mutations to serine. We depleted endogenous TCOF1 in nNOS-overexpressing Huh6 cells. After transfecting cells with wild-type (TCOF1-WT) or mutant vectors (C38S, C644S), biotin switch assays showed nNOS overexpression enhanced S-nitrosylation in TCOF1-WT and C38S, but not in C644S, indicating Cys644 is crucial for TCOF1 S-nitrosylation (Fig. 6D). Cell proliferation assays in TCOF1-WT and TCOF1-C644S cells revealed the C644S mutant restored cell proliferation inhibited by nNOS, as shown by CCK8, EdU and Ki67 fluorescence (Fig. 6E–G). In xenograft models, TCOF1-C644S tumors grew faster and were heavier than TCOF1-WT tumors. These findings suggest S-nitrosylation of TCOF1 at C644 is a novel mechanism for nNOS-mediated HB cell proliferation inhibition (Fig. 6H–J).

### 3.7 GSNOR rescues nNOS-Mediated Proliferation Suppression in HB via TCOF1 de-S-nitrosylation

**Fig. 7:**
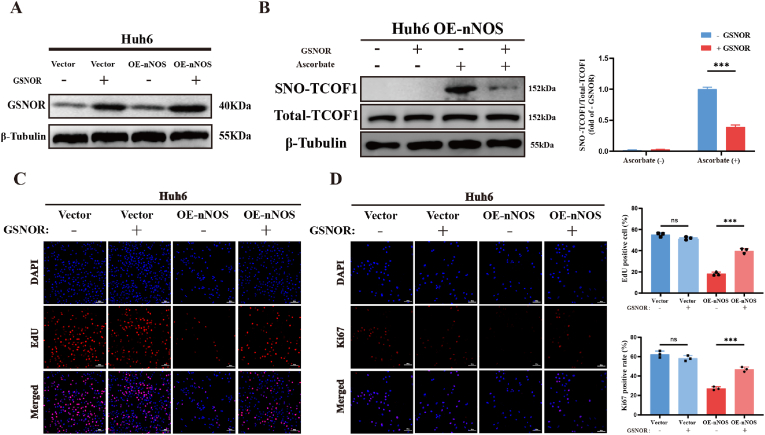
GSNOR Rescues nNOS-Mediated Proliferation Suppression in HB via TCOF1 De-S-nitrosylation. (A) Western blot showing endogenous GSNOR levels in parental and OE-nNOS Huh6 cells and confirming GSNOR overexpression. (B) Biotin-switch assay detecting SNO-TCOF1 in OE-nNOS Huh6 cells ± GSNOR overexpression; total TCOF1 blot is shown below. A lane without ascorbate served as the negative control. β-Tubulin: loading control; densitometry on the right. (C) EdU incorporation (scale bar, 100 μm) and (D) IF (scale bar, 50 μm) evaluating proliferation in Huh6 cells ± nNOS and ± GSNOR overexpression. Data are mean ± SD (n = 3 independent experiments). Statistics: Two-way ANOVA; ∗P < 0.05, ∗∗P < 0.01, ∗∗∗P < 0.001; ns, not significant.

GSNOR (S-nitrosoglutathione reductase) is a key de-S-nitrosylating enzyme that indirectly balances protein S-nitrosylation by regulating S-nitrosoglutathione (GSNO) levels [39]. To test whether GSNOR triggers TCOF1 de-S-nitrosylation in nNOS-overexpressing Huh6 cells, we quantified endogenous GSNOR in parental and OE-nNOS Huh6 cells and established a GSNOR overexpression system (Fig. 7A). Biotin-switch assays demonstrated that GSNOR overexpression sharply lowered TCOF1 S-nitrosylation in OE-nNOS cells (Fig. 7B). To determine how this modification affects nNOS-mediated suppression of HB proliferation, we performed EdU incorporation and Ki67 IF. GSNOR overexpression alone did not alter Huh6 proliferation, yet it significantly relieved the nNOS-induced growth arrest (Fig. 7C–D). Thus, GSNOR dampens the anti-proliferative effect of nNOS by decreasing TCOF1 S-nitrosylation.

### 3.8 nNOS Inhibits the Interaction Between TCOF1 and KRAS through the S-nitrosylation of TCOF1

**Fig. 8:**
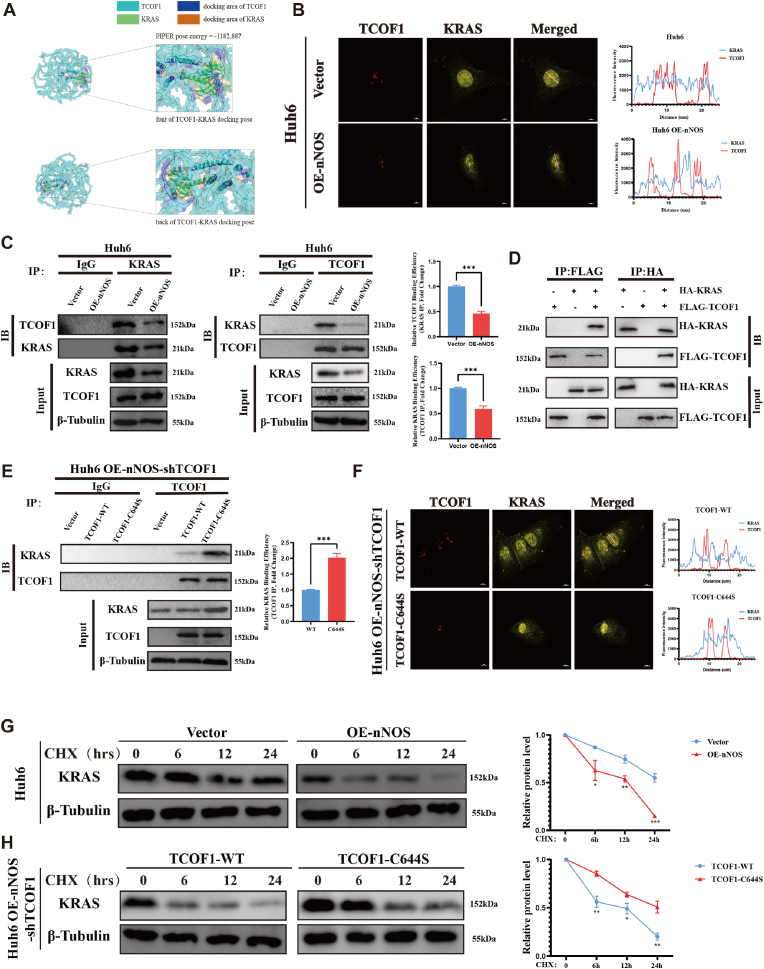
nNOS Inhibits the Interaction Between TCOF1 and KRAS Through the S-Nitrosylation of TCOF1. (A) ClusPro software (version 2.0), was used for molecular docking of TCOF1 and KRAS protein structures. TCOF1 (UniProt: Q13428) and KRAS (UniProt: P01116) protein structures were obtained from the UniProt database and modeled using AlphaFold2. The PIPER energy score was −1182.887. (B) IF analysis showing co-localization of TCOF1 (red) and KRAS (yellow) in Huh6 cells with or without nNOS overexpression. Nuclei were counterstained with DAPI (blue). Intensity profiles of indicated proteins along the plotted lines, as analyzed by ImageJ line scan analysis. Confocal imaging results are representative of three independent experiments. (C) Co-IP analysis of the endogenous interaction between TCOF1 and KRAS in Huh6 cells. Cell lysates were immunoprecipitated (IP) with KRAS or TCOF1 antibodies, followed by immunoblotting (IB) with reciprocal antibodies. IgG served as a negative control. (Right) Quantification of Co-IP binding efficiency. (D) Co-IP analysis of the exogenous interaction between HIS-tagged TCOF1 and HA-tagged KRAS in HK93T cells. Cells were co-transfected with FLAG-TCOF1 and HA-KRAS, and immunoprecipitated using FLAG or HA antibodies. The interaction between TCOF1 and KRAS was confirmed by reciprocal Co-IP. (E) Co-IP analysis of TCOF1-KRAS interaction nNOS-overexpressing Huh6 cells transfected with either TCOF1-WT or TCOF1-C644S mutant after TCOF1 knockout. (Right) Quantification of Co-IP binding efficiency. (F) IF analysis of TCOF1 (red) and KRAS (yellow) co-localization in nNOS-overexpressing Huh6 cells transfected with either TCOF1-WT or TCOF1-C644S mutant after TCOF1 knockout. Nuclei were counterstained with DAPI (blue). Protein intensity distribution along the line scan is indicated. (F) Analysis of protein stability following cycloheximide (CHX, 50ug/ml) treatment in Huh6 cells with nNOS overexpression compared to their vector control cells (upper panel). Analysis of protein stability following CHX treatment in nNOS-overexpressing Huh6 cells transfected with either TCOF1-WT or TCOF1-C644S mutant after TCOF1 knockout. (lower panel). Co-IP binding efficiency = [IP-Prey (experimental) × Input-Bait (control) × Input-Prey (control)] ÷ [IP-Prey (control) × Input-Bait (experimental) × Input-Prey (experimental)]. Relative Binding Efficiency = (Co-IP binding efficiency experimental) ÷ (Co-IP binding efficiency control). All experiments were performed in three independent replicates. Statistical analyses were performed using Student's t-test and two-way ANOVA. Error bars represent mean ± SD from triplicate samples (unless otherwise stated). Statistical significance was defined as ∗P < 0.05, ∗∗P < 0.01, ∗∗∗P < 0.001. scale bar, 10 μm.

**Fig. 9:**
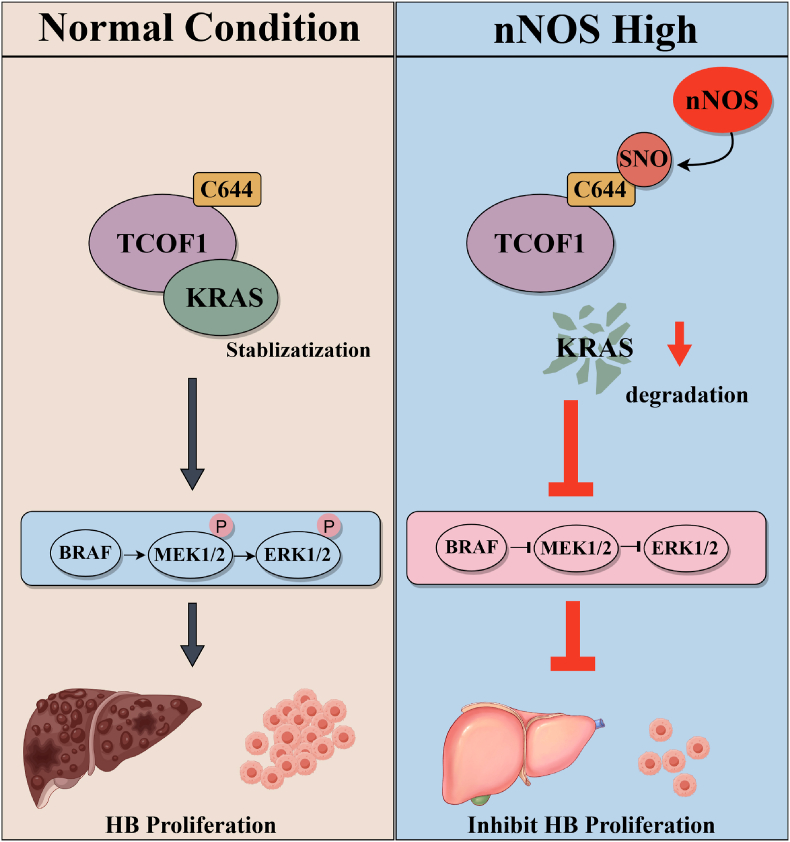
The pathway diagram illustrates nNOS-mediated S-nitrosylation of TCOF1 regulating KRAS proteostasis to suppress HB progression.

Our prior research revealed that nNOS not only markedly suppresses KRAS protein level but also induces S-nitrosylation of TCOF1. Since TCOF1 is documented to drive hepatocellular carcinoma by modulating KRAS activation genes [38], we posit that TCOF1 S-nitrosylation may impact KRAS expression via a defined molecular mechanism. To probe this, we performed molecular docking of TCOF1 and KRAS using ClusPro and generated high-precision 3D structural models with AlphaFold2 [40,41]. The analysis demonstrated a robust interaction between TCOF1 and KRAS, indicative of a stable protein complex (Fig. 8A). We systematically verified the KRAS–TCOF1 interaction. IF revealed pronounced nuclear co-localization of KRAS and TCOF1 in Huh6 cells (Fig. 8B). Co-IP corroborated this association and showed that nNOS markedly attenuates it (Fig. 8C). Reciprocal Co-IP in HEK293T cells co-expressing HA-KRAS and FLAG-TCOF1 confirmed the direct interaction (Fig. 8D). Importantly, nNOS overexpression diminished the interaction and reduced nuclear co-localization signal intensity; in contrast, the TCOF1-C644S mutant restored both parameters (Fig. 8E and F). These data indicate that nNOS-induced S-nitrosylation of TCOF1 at C644 disrupts TCOF1 binding to KRAS. Cycloheximide-chase assays demonstrated accelerated KRAS degradation upon nNOS overexpression in Huh6 cells (Fig. 8G). Re-expression of TCOF1-C644S in TCOF1-knockout, nNOS-overexpressing cells partially reversed this acceleration, whereas TCOF1-WT sustained the enhanced degradation (Fig. 8H). Collectively, nNOS-mediated S-nitrosylation of TCOF1 at C644 weakens KRAS–TCOF1 interaction, leading to reduced KRAS stability and facilitating its proteolysis (see Fig. 9).

## 4 Discussion

Despite advances in multimodal therapies for HB, patients with advanced-stage disease face dismal survival rates due to chemotherapy resistance and metastatic progression. Our study identifies nNOS as a tumor suppressor in HB, offering a novel therapeutic axis targeting the nNOS-TCOF1-KRAS pathway—a mechanism distinct from current strategies.

Although the role of nNOS in tumors is gaining attention, research in this area is still limited [42,43]. Our data demonstrate that the expression of nNOS is lower in HB tissues compared to normal liver tissues. Furthermore, the low expression of nNOS is closely associated with the clinical staging of HB, suggesting a negative regulatory role of nNOS in the development of HB. By establishing stable overexpression of nNOS in HB cell lines and measuring its effects on cell proliferation and apoptosis, this study is the first to confirm that nNOS serves as a negative regulator of HB proliferation.

Earlier research findings suggest that 2-methoxyestradiol induces enzyme uncoupling by elevating nNOS protein levels, resulting in excessive accumulation of reactive nitrogen species that trigger pro-apoptotic responses in metastatic osteosarcoma [16]. Additionally, docosahexaenoic acid promotes the accumulation of intracellular NO and ROS concentrations by activating nNOS, leading to induction of apoptosis in cancer cells [44]. Overexpression of nNOS in K562 cells of chronic myeloid leukemia inhibits the PI3K/Akt/mTORC signaling pathway while activating the JNK/p38 MAPK/ERK1/2 signaling pathway, ultimately promoting apoptosis [45]. While nNOS exhibits context-dependent roles in cancer, our work is the first to demonstrate its tumor-suppressive function in HB. Unlike its pro-apoptotic effects in osteosarcoma or dual roles in leukemia [44,45], nNOS inhibits HB proliferation through S-nitrosylation of TCOF1, a post-translational modification previously unexplored in this malignancy. This divergence underscores the tissue-specificity of NO signaling and highlights TCOF1 as a redox-sensitive node in HB pathogenesis.

Research on the role of nNOS-regulated S-nitrosylation modifications in tumors remains sparse, with the majority of studies coming from our laboratory. Initially, we discovered that nNOS activates the Akt/mTOR pathway by inducing S-nitrosylation of PTEN, which in turn inhibits autophagy in nasopharyngeal carcinoma [24]. Subsequently, we demonstrated that nNOS induces the nuclear translocation of GAPDH via S-nitrosylation, thereby activating apoptotic responses in colon cancer [25]. Moreover, we found that nNOS enhances the S-nitrosylation of PFKM, which promotes glycolysis in ovarian cancer cells [26]. Furthermore, our study has, for the first time, demonstrated that nNOS inhibits HB cell proliferation through the S-nitrosylation of TCOF1 and elucidated the underlying mechanism. TCOF1 promotes the colorectal cancer progression by interacting with β-catenin and stabilizing its expression [46]. In the TCOF1 knockdown zebrafish model, a reduction in ribosomal RNA transcription, increased p53 stability, and enhanced cell death were observed [47]. TCOF1 coordinates rDNA transcription, oncogenic activation, and immune infiltration to drive the development of hepatocellular carcinoma [38]. Upregulation of TCOF1 in triple-negative breast cancer promotes stemness and tumor growth, correlating with poor prognosis [48]. However, no studies have yet investigated the molecular mechanisms by which TCOF1 promotes tumor progression from the perspective of post-translational modifications. Through S-nitrosylation proteomics, software prediction, and S-nitrosylation protein detection experiments, we discovered that nNOS S-nitrosylates TCOF1, which in turn disrupts the interaction between TCOF1 and KRAS, inhibits KRAS expression. Previous work shows that S-nitrosylation of KRAS at Cys118 enhances its GTPase activity and thereby activates MAPK signaling [[49], [50], [51]]. In contrast, our system reveals no direct S-nitrosylation of KRAS by nNOS; rather, nNOS S-nitrosylates TCOF1 at Cys644, disrupting the TCOF1-KRAS interaction and accelerating KRAS degradation.

Although KRAS mutations are infrequent in HB [52], aberrant KRAS expression drives pathogenesis, particularly in the embryonal subtype where KRAS-Wnt/β-catenin crosstalk enhances proliferation and chemoresistance [53]. Here, we uncover a redox-regulated mechanism of KRAS stabilization: nNOS deficiency permits TCOF1-KRAS interaction, sustaining MAPK activation and tumor progression. This non-mutational paradigm aligns with findings in KRAS-mutant cancers, where constitutive pathway activation escalates downstream signaling [54,55]. Critically, we demonstrate that TCOF1 S-nitrosylation—modulated by nNOS—serves as a molecular switch for KRAS degradation, offering a therapeutic strategy to circumvent mutation-driven resistance. Our work repositions TCOF1 as a universal chaperone for oncoprotein stabilization, with implications beyond HB.

KRAS stability is governed by a network of regulators: HSP90 ensures proper folding [56], USP7 removes K48 ubiquitin chains to prevent degradation [57], and lncRNA KIMAT1 scaffolds the HSP90-KRAS complex [58]. Here, we uncover a redox-dependent layer of control—TCOF1 stabilizes KRAS via direct binding, but nNOS-mediated S-nitrosylation of TCOF1 at C644 disrupts this interaction, thereby facilitating KRAS proteolysis. Unlike HSP90/USP7 targeting strategies that risk systemic toxicity, modulating this axis via nNOS could selectively deplete KRAS in tumors. However, two limitations remain: first, the structural and regulatory basis for nNOS-selective S-nitrosylation of TCOF1-C644 remains elusive; second, the precise degradation route triggered in KRAS after its release from TCOF1 is still unknown. Addressing these questions will be central to future work.

In summary, we redefine nNOS as a guardian of KRAS proteostasis in HB, coupling NO signaling to post-translational control of oncoprotein stability. By demonstrating that S-nitrosylation disrupts TCOF1-KRAS binding—a druggable protein-protein interaction—our work positions nNOS activation as a strategy to destabilize KRAS in malignancies where mutations are absent but pathway activation prevails.

## CRediT authorship contribution statement

**Meng Wang:** Writing – review & editing, Writing – original draft, Visualization, Validation, Supervision, Software, Project administration, Methodology, Investigation, Formal analysis, Data curation, Conceptualization. **Yupeng Wang:** Writing – review & editing, Writing – original draft, Visualization, Validation, Software, Methodology, Investigation, Formal analysis, Data curation, Conceptualization. **Yue Qian:** Writing – review & editing, Writing – original draft, Visualization, Validation, Software, Project administration, Methodology, Investigation, Formal analysis, Data curation, Conceptualization. **Ziyan Luo:** Writing – review & editing. **Siqi Dong:** Writing – review & editing. **zhuoyan Li:** Writing – review & editing. **Lingling Wu:** Writing – review & editing. **Fang Yu:** Writing – review & editing. **Zihua Lin:** Writing – review & editing. **Lin Qiu:** Writing – review & editing. **Hua Jiang:** Writing – review & editing. **Linna Yu:** Writing – review & editing, Writing – original draft, Visualization, Validation, Supervision, Software, Resources, Project administration, Methodology, Investigation, Funding acquisition, Formal analysis, Data curation, Conceptualization.

## Data transparency

The authors confirm that no data manipulation or AI-assisted writing tools were used.

## Funding

This work was supported by the 10.13039/501100001809National Natural Science Foundation of China (No. 82370172), 10.13039/501100021171Basic and Applied Basic Research Foundation of Guangdong Province (No. 2023A1515220005), Clinical Specialty Technology Program of Guangzhou Municipal Health Commission (No. 2023C-TS56), High-Level Talent Support Program of Yunnan Provincial Health Commission (No. 2023-KHRCBZ-B04), Basic and Applied Basic Research Project of Guangzhou Science and Technology Bureau (No. 2023A04J1894).

## Declaration of competing interest

The authors declare that they have no known competing financial interests or personal relationships that could have appeared to influence the work reported in this paper.

## Data Availability

Data will be made available on request.

## Appendix A Supplementary data

Multimedia component 1 Fig. S1. nNOS Overexpression Inhibits HB Cell Proliferation In Vitro. (A) Confirmation of nNOS protein expression in HepG2 cells via Western blot and IF microscopy (GFP, green) (scale bar, 100 μm). Cells were transfected with either an empty vector (vector group) or an nNOS lentiviral overexpression vector (OE-nNOS group). (B) EdU staining (scale bar, 100 μm), (C) CCK8 assay, and (D) Ki67 IF (scale bar, 50 μm) assessed the proliferative capacity of nNOS-overexpressing HepG2 cells versus controls. Quantification of EdU- and Ki67-positive cells is shown on the right. (E) EdU staining verified the proliferation of vector and OE-nNOS HepG2 cell groups treated with 1 μM l-NAME, 2 μM 1400W, and 2 μM inNOS (scale bar, 100 μm). Quantification of EdU staining results is shown on the right. Data are presented as mean ± SD from three independent experiments. Statistical significance was determined using independent t-tests (∗P < 0.05, ∗∗P < 0.01, ∗∗∗P < 0.001; ns, not significant). Fig. S2. iNOS Overexpression Enhances HB Cell Proliferation In Vitro. (A) Western blot analysis was conducted to evaluate iNOS levels in Huh6 cells, as well as to confirm iNOS overexpression. (B) CCK8 assay was utilized to evaluate the proliferative capacity of Huh6 cells with iNOS overexpression compared to their vector control cells. (C) EdU staining was performed to verify the proliferation of vector and OE-iNOS Huh6 cell groups, each treated with 1 μM l-NAME, 2 μM 1400W and 2 μM inNOS (scale bar, 100 μm). (D) Quantification of EdU staining results. The results are shown as the mean ± SD obtained from three independent experiments. Statistical analysis was performed using independent t-tests and two-way ANOVA. Statistical significance was defined as ∗P < 0.05, ∗∗P < 0.01, ∗∗∗P < 0.001. ns, not significant. Fig. S3. Transcriptomic and Proteomic Analysis Reveals nNOS Suppresses HB Cell Proliferation via MAPK Pathway Inhibition. (A) Principal component analysis (PCA) of RNA-seq data. (B) PCA of TMT-based proteomics data. (C) Gene Ontology (GO) enrichment analysis of transcriptomic differentially expressed genes.(D) GO analysis of proteomic differentially expressed proteins. Fig. S4. nNOS Overexpression Suppresses KRAS to Regulate MAPK Signaling. (A) Relative mRNA expression levels of MAPK pathway-related genes, including HRAS, KRAS, NRAS, ARAF, BRAF, CRAF, MEK1/2, and ERK1/2, in HepG2 cells transfected with nNOS overexpression plasmid (OE-nNOS) or vector control (Vector). (B) Western blot analysis of the protein expression levels of MAPK pathway components, including HRAS, KRAS, NRAS, ARAF, BRAF, CRAF, MEK1/2, p-MEK1/2, ERK1/2, and p-ERK1/2, in the same experimental groups. β-Tubulin served as a loading control. Representative blots (left) and corresponding quantified data (right) are shown. (C–F) IF staining of KRAS (C), BRAF (D), MEK1/2 and p-MEK1/2 (E), ERK1/2 and p-ERK1/2 (F) in the same experimental groups. Representative images of these indicators with DAPI-stained nuclei (blue) are shown, along with quantified fluorescence intensity (right panels). Data are presented as mean ± SD from three independent experiments. Statistical analysis was performed using Student's *t*-test. Significance levels are indicated as ∗P < 0.05, ∗∗P < 0.01, ∗∗∗P < 0.001; ns, not significant. Scale bar, 50 μm. Fig. S5. Effects of nNOS Overexpression on Subcellular Localization and NO Production in HB Cells. (A) Representative IF images showing the localization of nNOS in Huh6 cells with and without nNOS overexpression (scale bar, 5 μm). (B) Intracellular NO levels detected in Vector and OE-nNOS cells using the NO-specific fluorescent probe BBoxiProbe® O38 (scale bar, 50 μm). (C) Total NO metabolites determined in Vector and OE-nNOS cells using an improved Griess method, reflecting intracellular NO content. (D) DHE fluorescence intensity reflecting intracellular O_2_^−^ levels detected in Vector, OE-nNOS cells, and positive control cells treated with Rosup II (scale bar, 50 μm). Data are presented as mean ± SD from three independent experiments. Statistical analysis was performed using Student's *t*-test. Significance levels are indicated as ∗P < 0.05, ∗∗P < 0.01, ∗∗∗P < 0.001. Fig. S6. Global Analysis of Protein S-Nitrosylation Mediated by nNOS in Huh6 cells. (A) RSD showed the sample reproducibility in S-nitrosylation Proteomics. (B) PCA plot demonstrates a significant separation between nNOS overexpression samples and control group samples in the principal component space. (C) Quality control checks evaluated the distribution of peptide lengths and charges. (D) A heatmap displaying the relative expression levels of multiple differential S-nitrosylation-modified sites in Huh6 cells overexpressing nNOS compared to control cells. Red represents high expression, green indicates low expression. (E-F) GO Molecular Function and Cellular Component enrichment analysis revealed various functional categories potentially associated with S-nitrosylation modifications. Fig. S7. nNOS-Specific S-Nitrosylation of TCOF1 (A) Co-IP analysis was conducted to investigate the interaction between nNOS and KRAS or TCOF1 in Huh6 cells overexpressing nNOS. Cell lysates were IP using nNOS antibodies, followed by IB with KRAS and TCOF1 antibodies. IgG was used as a negative control. (B) Detection of SNO-TCOF1 in protein extracts from OE-iNOS and vector Huh6 cells upon biotin switch assay, followed by Total-TCOF1 Western blot. No ascorbate was added as a negative control. β-Tubulin was used as a loading control. Densitometry is shown on the right. All experiments were conducted in three independent replicates. Multimedia component 1

## References

[1] Kahla J.A., Siegel D.A., Dai S., Lupo P.J., Foster J.H., Scheurer M.E. (2022). Incidence and 5-year survival of children and adolescents with hepatoblastoma in the United States. Pediatr. Blood Cancer.

[2] Meyers R.L., Maibach R., Hiyama E., Haberle B., Krailo M., Rangaswami A. (2017). Risk-stratified staging in paediatric hepatoblastoma: a unified analysis from the Children's Hepatic tumors International Collaboration. Lancet Oncol..

[3] Kluiver T.A., Lu Y., Schubert S.A., Kraaier L.J., Ringnalda F., Lijnzaad P. (2024). Divergent WNT signaling and drug sensitivity profiles within hepatoblastoma tumors and organoids. Nat. Commun..

[4] Failli M., Demir S., Del Rio-Alvarez A., Carrillo-Reixach J., Royo L., Domingo-Sabat M. (2024). Computational drug prediction in hepatoblastoma by integrating pan-cancer transcriptomics with pharmacological response. Hepatology.

[5] Espinoza A.F., Patel R.H., Patel K.R., Badachhape A.A., Whitlock R., Srivastava R.K. (2024). A novel treatment strategy utilizing panobinostat for high-risk and treatment-refractory hepatoblastoma. J. Hepatol..

[6] Fang J., Singh S., Cheng C., Natarajan S., Sheppard H., Abu-Zaid A. (2023). Genome-wide mapping of cancer dependency genes and genetic modifiers of chemotherapy in high-risk hepatoblastoma. Nat. Commun..

[7] Semeraro M., Branchereau S., Maibach R., Zsiros J., Casanova M., Brock P. (2013). Relapses in hepatoblastoma patients: clinical characteristics and outcome--experience of the international childhood liver tumour strategy group (SIOPEL). Eur. J. Cancer.

[8] Wu J., Zhou Z., Li J., Liu H., Zhang H., Zhang J. (2023). CHD4 promotes acquired chemoresistance and tumor progression by activating the MEK/ERK axis. Drug Resist. Updates.

[9] Huang H., Wu L., Lu L., Zhang Z., Qiu B., Mo J. (2023). Single-cell transcriptomics uncovers cellular architecture and developmental trajectories in hepatoblastoma. Hepatology.

[10] Roehrig A., Hirsch T.Z., Pire A., Morcrette G., Gupta B., Marcaillou C. (2024). Single-cell multiomics reveals the interplay of clonal evolution and cellular plasticity in hepatoblastoma. Nat. Commun..

[11] Drehmer D., Mesquita Luiz J.P., Hernandez C.A.S., Alves-Filho J.C., Hussell T., Townsend P.A. (2022). Nitric oxide favours tumour-promoting inflammation through mitochondria-dependent and -independent actions on macrophages. Redox Biol..

[12] O'Gallagher K., Rosentreter R.E., Elaine Soriano J., Roomi A., Saleem S., Lam T. (2022). The effect of a neuronal nitric oxide synthase inhibitor on neurovascular regulation in humans. Circ. Res..

[13] Ding Y., Jin Y., Peng T., Gao Y., Zang Y., He H. (2022). Fabrication of multifunctional metal-organic frameworks nanoparticles via layer-by-layer self-assembly to efficiently discover PSD95-nNOS uncouplers for stroke treatment. J. Nanobiotechnol..

[14] Kumar A., Goel H.L., Wisniewski C.A., Wang T., Geng Y., Wang M. (2024). Neuropilin-2-expressing breast cancer cells mitigate radiation-induced oxidative stress through nitric oxide signaling. J. Clin. Investig..

[15] Zou Q., Zhou X., Lai J., Zhou H., Su J., Zhang Z. (2025). Targeting p62 by sulforaphane promotes autolysosomal degradation of SLC7A11, inducing ferroptosis for osteosarcoma treatment. Redox Biol..

[16] Gorska-Ponikowska M., Ploska A., Jacewicz D., Szkatula M., Barone G., Lo Bosco G. (2020). Modification of DNA structure by reactive nitrogen species as a result of 2-methoxyestradiol-induced neuronal nitric oxide synthase uncoupling in metastatic osteosarcoma cells. Redox Biol..

[17] Zou Z., Li X., Sun Y., Li L., Zhang Q., Zhu L. (2020). NOS1 expression promotes proliferation and invasion and enhances chemoresistance in ovarian cancer. Oncol. Lett..

[18] Mattioli E.J., Rossi J., Meloni M., De Mia M., Marchand C.H., Tagliani A. (2022). Structural snapshots of nitrosoglutathione binding and reactivity underlying S-nitrosylation of photosynthetic GAPDH. Redox Biol..

[19] Shi X., O'Connor M., Qiu H. (2024). Valosin-containing protein acts as a target and mediator of S-nitrosylation in the heart through distinct mechanisms. Redox Biol..

[20] Tang X., Pan L., Zhao S., Dai F., Chao M., Jiang H. (2020). SNO-MLP (S-nitrosylation of Muscle LIM protein) facilitates myocardial hypertrophy through TLR3 (Toll-Like Receptor 3)-mediated RIP3 (Receptor-Interacting protein Kinase 3) and NLRP3 (NOD-Like receptor Pyrin Domain containing 3) Inflammasome activation. Circulation.

[21] Chao M.L., Luo S., Zhang C., Zhou X., Zhou M., Wang J. (2021). S-nitrosylation-mediated coupling of G-protein alpha-2 with CXCR5 induces Hippo/YAP-dependent diabetes-accelerated atherosclerosis. Nat. Commun..

[22] Fonseca F.V., Raffay T.M., Xiao K., McLaughlin P.J., Qian Z., Grimmett Z.W. (2022). S-nitrosylation is required for beta(2)AR desensitization and experimental asthma. Mol Cell.

[23] Andreyev A.Y., Yang H., Doulias P.T., Dolatabadi N., Zhang X., Luevanos M. (2024). Metabolic Bypass Rescues aberrant S-nitrosylation-Induced TCA cycle inhibition and synapse loss in Alzheimer's disease human neurons. Adv. Sci. (Weinh.).

[24] Zhu L., Li L., Zhang Q., Yang X., Zou Z., Hao B. (2017). NOS1 S-nitrosylates PTEN and inhibits autophagy in nasopharyngeal carcinoma cells. Cell Death Discov..

[25] Li K., Huang M., Xu P., Wang M., Ye S., Wang Q. (2020). Microcystins-LR induced apoptosis via S-nitrosylation of GAPDH in colorectal cancer cells. Ecotoxicol. Environ. Saf..

[26] Gao W., Huang M., Chen X., Chen J., Zou Z., Li L. (2021). The role of S-nitrosylation of PFKM in regulation of glycolysis in ovarian cancer cells. Cell Death Dis..

[27] Nie X., Xiao D., Ge Y., Xie Y., Zhou H., Zheng T. (2021). TRF2 recruits nucleolar protein TCOF1 to coordinate telomere transcription and replication. Cell Death Differ..

[28] Calo E., Gu B., Bowen M.E., Aryan F., Zalc A., Liang J. (2018). Tissue-selective effects of nucleolar stress and rDNA damage in developmental disorders. Nature.

[29] He X., Zhao J., Adilijiang A., Hong P., Chen P., Lin X. (2023). Dhx33 promotes B-cell growth and proliferation by controlling activation-induced rRNA upregulation. Cell. Mol. Immunol..

[30] Zhang L., Ren B.C., Wei F., Liu Y., Gao Y., Yuan B. (2023). Ferroptosis regulator NOS2 is closely associated with the prognosis and cell malignant behaviors of hepatoblastoma: a bioinformatic and in vitro study. Front. Oncol..

[31] Guo T., Steen J.A., Mann M. (2025). Mass-spectrometry-based proteomics: from single cells to clinical applications. Nature.

[32] Carnicer R., Suffredini S., Liu X., Reilly S., Simon J.N., Surdo N.C. (2017). The subcellular localisation of neuronal nitric oxide synthase determines the downstream effects of NO on Myocardial function. Cardiovasc. Res..

[33] Yao Y., Hu Y., Yang J., Zhang C., He Y., Qi H. (2022). Inhibition of neuronal nitric oxide synthase protects against hippocampal neuronal injuries by increasing neuropeptide Y expression in temporal lobe epilepsy mice. Free Radic. Biol. Med..

[34] Oh C.K., Nakamura T., Zhang X., Lipton S.A. (2024). Redox regulation, protein S-nitrosylation, and synapse loss in Alzheimer's and related dementias. Neuron.

[35] Bohle F., Rossi J., Tamanna S.S., Jansohn H., Schlosser M., Reinhardt F. (2024). Chloroplasts lacking class I glutaredoxins are functional but show a delayed recovery of protein cysteinyl redox state after oxidative challenge. Redox Biol..

[36] Yin Y.L., Chen Y., Ren F., Wang L., Zhu M.L., Lu J.X. (2022). Nitrosative stress induced by homocysteine thiolactone drives vascular cognitive impairments via GTP cyclohydrolase 1 S-nitrosylation in vivo. Redox Biol..

[37] Stobernack T., Dommershausen N., Alcolea-Rodriguez V., Ledwith R., Banares M.A., Haase A. (2024). Advancing nanomaterial toxicology screening through efficient and cost-effective quantitative proteomics. Small Methods.

[38] Wu C., Xia D., Wang D., Wang S., Sun Z., Xu B. (2022). TCOF1 coordinates oncogenic activation and rRNA production and promotes tumorigenesis in HCC. Cancer Sci..

[39] Rizza S., Filomeni G. (2017). Chronicles of a reductase: biochemistry, genetics and physio-pathological role of GSNOR. Free Radic. Biol. Med..

[40] Jeppesen M., Andre I. (2023). Accurate prediction of protein assembly structure by combining AlphaFold and symmetrical docking. Nat. Commun..

[41] Jimenez-Garcia B., Roel-Touris J., Barradas-Bautista D. (2023). The LightDock server: artificial intelligence-powered modeling of macromolecular interactions. Nucleic Acids Res..

[42] Zhang Y., Liao Q., Wen X., Fan J., Yuan T., Tong X. (2025). Hijacking of the nervous system in cancer: mechanism and therapeutic targets. Mol. Cancer.

[43] Tang X., Li K., Wang Y., Rocchi S., Shen S., Cerezo M. (2024). Metabolism and mRNA translation: a nexus of cancer plasticity. Trends Cell Biol..

[44] Akimov M.G., Gamisonia A.M., Dudina P.V., Gretskaya N.M., Gaydaryova A.A., Kuznetsov A.S. (2021). GPR55 receptor activation by the N-Acyl Dopamine family Lipids induces apoptosis in cancer cells via the Nitric Oxide Synthase (nNOS) over-Stimulation. Int. J. Mol. Sci..

[45] Sadaf S., Awasthi D., Singh A.K., Nagarkoti S., Kumar S., Barthwal M.K. (2020). Pyroptotic and apoptotic cell death in iNOS and nNOS overexpressing K562 cells: a mechanistic insight. Biochem. Pharmacol..

[46] Yun H., You J.E., Hong J.K., Kim D.Y., Lee J.U., Kang D.H. (2023). TCOF1 promotes the colorectal cancer progression by stabilizing beta-catenin. Med. Oncol..

[47] de Peralta M.S., Mouguelar V.S., Sdrigotti M.A., Ishiy F.A., Fanganiello R.D., Passos-Bueno M.R. (2016). Cnbp ameliorates Treacher Collins Syndrome craniofacial anomalies through a pathway that involves redox-responsive genes. Cell Death Dis..

[48] Hu J., Lai Y., Huang H., Ramakrishnan S., Pan Y., Ma V.W.S. (2022). TCOF1 upregulation in triple-negative breast cancer promotes stemness and tumour growth and correlates with poor prognosis. Br. J. Cancer.

[49] Batista W.L., Ogata F.T., Curcio M.F., Miguel R.B., Arai R.J., Matsuo A.L. (2013). S-nitrosoglutathione and endothelial nitric oxide synthase-derived nitric oxide regulate compartmentalized ras S-nitrosylation and stimulate cell proliferation. Antioxidants Redox Signal..

[50] Kramer-Drauberg M., Ambrogio C. (2021). Discoveries in the redox regulation of KRAS. Int. J. Biochem. Cell Biol..

[51] Huang L., Carney J., Cardona D.M., Counter C.M. (2014). Decreased tumorigenesis in mice with a Kras point mutation at C118. Nat. Commun..

[52] Martin-Giacalone B.A., Li H., Scheurer M.E., Casey D.L., Dugan-Perez S., Marquez-Do D.A. (2024). Germline genetic testing and survival outcomes among children with Rhabdomyosarcoma: a report from the children's oncology group. JAMA Netw. Open.

[53] Nagae G., Yamamoto S., Fujita M., Fujita T., Nonaka A., Umeda T. (2021). Genetic and epigenetic basis of hepatoblastoma diversity. Nat. Commun..

[54] Huang L., Guo Z., Wang F., Fu L. (2021). KRAS mutation: from undruggable to druggable in cancer. Signal Transduct. Targeted Ther..

[55] Fey S.K., Najumudeen A.K., Watt D.M., Millett L.M., Ford C.A., Gilroy K. (2025). KRAS loss of Heterozygosity promotes MAPK-dependent pancreatic ductal adenocarcinoma initiation and induces therapeutic sensitivity to MEK inhibition. Cancer Res..

[56] Ostrem J.M., Peters U., Sos M.L., Wells J.A., Shokat K.M. (2013). K-Ras(G12C) inhibitors allosterically control GTP affinity and effector interactions. Nature.

[57] Huang B., Cao D., Yuan X., Xiong Y., Chen B., Wang Y. (2024). USP7 deubiquitinates KRAS and promotes non-small cell lung cancer. Cell Rep..

[58] Shi L., Magee P., Fassan M., Sahoo S., Leong H.S., Lee D. (2021). A KRAS-responsive long non-coding RNA controls microRNA processing. Nat. Commun..

