# Supplementary material for: nNOS-mediated S-nitrosylation of TCOF1 regulates KRAS proteostasis to suppress hepatoblastoma progression
Source: Redox Biol. 2025 Sep 20;87:103870. doi: 10.1016/j.redox.2025.103870 (PMC12552980; doi:10.1016/j.redox.2025.103870)
Supplement: Multimedia component 1 — Fig. S1. nNOS Overexpression Inhibits HB Cell Proliferation In Vitro. (A) Confirmation of nNOS protein expression in HepG2 cells via Western blot and IF microscopy (GFP, green) (scale bar, 100 μm). Cells were transfected with either an empty vector (vector group) or an nNOS lentiviral overexpression vector (OE-nNOS group). (B) EdU staining (scale bar, 100 μm), (C) CCK8 assay, and (D) Ki67 IF (scale bar, 50 μm) assessed the proliferative capacity of nNOS-overexpressing HepG2 cells versus controls. Quantification of EdU- and Ki67-positive cells is shown on the right. (E) EdU staining verified the proliferation of vector and OE-nNOS HepG2 cell groups treated with 1 μM l-NAME, 2 μM 1400W, and 2 μM inNOS (scale bar, 100 μm). Quantification of EdU staining results is shown on the right. Data are presented as mean ± SD from three independent experiments. Statistical significance was determined using independent t-tests (∗P < 0.05, ∗∗P < 0.01, ∗∗∗P < 0.001; ns, not significant). Fig. S2. iNOS Overexpression Enhances HB Cell Proliferation In Vitro. (A) Western blot analysis was conducted to evaluate iNOS levels in Huh6 cells, as well as to confirm iNOS overexpression. (B) CCK8 assay was utilized to evaluate the proliferative capacity of Huh6 cells with iNOS overexpression compared to their vector control cells. (C) EdU staining was performed to verify the proliferation of vector and OE-iNOS Huh6 cell groups, each treated with 1 μM l-NAME, 2 μM 1400W and 2 μM inNOS (scale bar, 100 μm). (D) Quantification of EdU staining results. The results are shown as the mean ± SD obtained from three independent experiments. Statistical analysis was performed using independent t-tests and two-way ANOVA. Statistical significance was defined as ∗P < 0.05, ∗∗P < 0.01, ∗∗∗P < 0.001. ns, not significant. Fig. S3. Transcriptomic and Proteomic Analysis Reveals nNOS Suppresses HB Cell Proliferation via MAPK Pathway Inhibition. (A) Principal component analysis (PCA) of RNA-seq data. (B) PCA of TMT- [file mmc1.pdf]

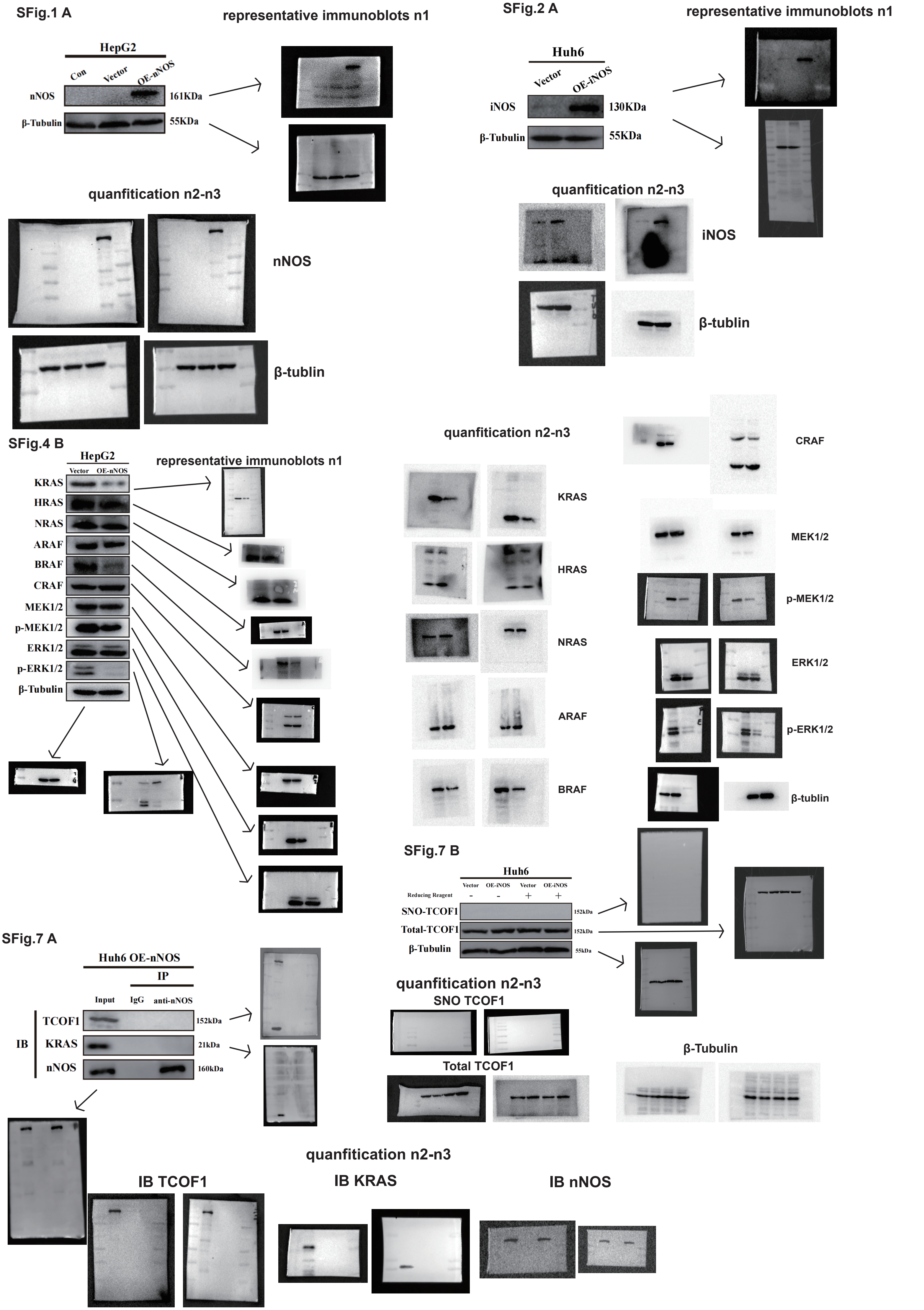
SFig.1 A
representative immunoblots n1
HepG2
Con
Vector
OE-nNOS
nNOS
161KDa
β-Tubulin
55KDa
quanfitication n2-n3
nNOS
β-tublin
SFig.2 A
representative immunoblots n1
Huh6
Vector
OE-iNOS
iNOS
130KDa
β-Tubulin
55KDa
quanfitication n2-n3
iNOS
β-tublin
SFig.4 B
HepG2
Vector
OE-nNOS
KRAS
HRAS
NRAS
ARAF
BRAF
CRAF
MEK1/2
p-MEK1/2
ERK1/2
p-ERK1/2
β-Tubulin
representative immunoblots n1
quanfitication n2-n3
KRAS
HRAS
NRAS
ARAF
BRAF
CRAF
MEK1/2
p-MEK1/2
ERK1/2
p-ERK1/2
β-tublin
SFig.7 B
Huh6
Vector
OE-iNOS
Vector
OE-iNOS
Reducing Reagent
-
-
+
+
SNO-TCOF1
152kDa
Total-TCOF1
152kDa
β-Tubulin
55kDa
quanfitication n2-n3
SNO TCOF1
Total TCOF1
β-Tubulin
SFig.7 A
Huh6 OE-nNOS
IP
Input
IgG
anti-nNOS
IB
TCOF1
152kDa
KRAS
21kDa
nNOS
160kDa
quanfitication n2-n3
IB TCOF1
IB KRAS
IB nNOS
