# Supplementary material for: nNOS-mediated S-nitrosylation of TCOF1 regulates KRAS proteostasis to suppress hepatoblastoma progression
Source: Redox Biol. 2025 Sep 20;87:103870. doi: 10.1016/j.redox.2025.103870 (PMC12552980; doi:10.1016/j.redox.2025.103870)
Supplement: Multimedia component 2 [file mmc2.docx]

**Table S1. Antibodies used in this study**

| **Antibodys** | **Source** | **Catalog#** | **Dilution** |
| --- | --- | --- | --- |
| nNOS | Abcam | ab76067 | 1:2000(WB)  1:200(IHC)  1:200(IF) |
| Ki67 | HUABIO | HA721115 | 1:500(IHC) |
| Midkine | HUABIO | ET1702-64 | 1:200(IHC) |
| iNOS | CST | 13120 | 1:2000(WB) |
| KRAS（mouse） | HUABIO | HA601059 | 1:2000(WB)1:200 |
| KRAS（rabbit） | Proteintech | 12063-1-AP | 1:2000(WB)  1:200(IF)  1:100(IP) |
| TCOF1 | Proteintech | 11003-1-AP | 1:2000(WB)  1:200(IF)  1:100(IP) |
| GSNOR | Proteintech | 11051-1-AP | 1:2000(WB) |
| HA-tag | HUABIO | 0906-1 | 1:2000(WB) |
| DYKDDDDK Tag | HUABIO | 0912-1 | 1:2000(WB) |
| β-Tubulin | HUABIO | M1305-2 | 1:2000(WB) |
| ARAF | HUABIO | ET1701-18 | 1:2000(WB)1:200(IF) |
| BRAF | HUABIO | ET1608-36 | 1:2000(WB)1:200(IF) |
| CRAF | HUABIO | ET1701-21 | 1:2000(WB)1:200(IF) |
| HRAS | HUABIO | 15531-1-AP | 1:2000(WB)1:200(IF) |
| NRAS | HUABIO | 10724-1-AP | 1:2000(WB)1:200(IF) |
| ERK1/2 | Proteintech | 66192-1-Ig | 1:2000(WB)1:100(IF) |
| Phospho-ERK1/2 | Proteintech | 28733-1-AP | 1:2000(WB)1:100(IF) |
| MEK1/2 | Proteintech | 11049-1-AP | 1:2000(WB)1:100(IF) |
| Phospho-MEK1/2 | CST | 2338 | 1:2000(WB)1:100(IF) |
| BAX | Proteintech | 50599-2-Ig | 1:2000(WB) |
| BCL-2 | Proteintech | 12789-1-AP | 1:2000(WB) |
| Caspase-3 | Santa Cruz Biotechnology | sc-56046 | 1:2000(WB) |
| Rabbit IgG | CST | 2729 | 1:100（IP） |
| IgG H&L (HRP) | Abcam | ab205718 | 1:500(IHC) |
| HRP AffiniPure Goat Anti-Mouse IgG（H+L） | Fudebio | FDM007 | 1:10000  (WB) |
| HRP AffiniPure Goat Anti-Rabbit IgG（H+L） | Fudebio | FDR007 | 1:10000  (WB) |
| DyLight 649 AffiniPure Goat Anti-Rabbit IgG | Fudebio | FD0130 | 1:500(IHC) |
| DyLight 549 AffiniPure Goat Anti-Mouse IgG | Fudebio | FD0146 | 1:500(IHC) |
| Anti-rabbit IgG for IP (HRP) | Vazyme | RA1008-01 | 1：500（WB） |
| Anti-mouse IgG for IP (HRP) | Vazyme | RA1009-01 | 1：500（WB） |

WB/IF/IP/IHC,Western blot/Immunofluorescence/immunoprecipitation/immunohistochemistry.
