# Supplementary material for: nNOS-mediated S-nitrosylation of TCOF1 regulates KRAS proteostasis to suppress hepatoblastoma progression
Source: Redox Biol. 2025 Sep 20;87:103870. doi: 10.1016/j.redox.2025.103870 (PMC12552980; doi:10.1016/j.redox.2025.103870)
Supplement: Multimedia component 4 [file mmc4.pdf]

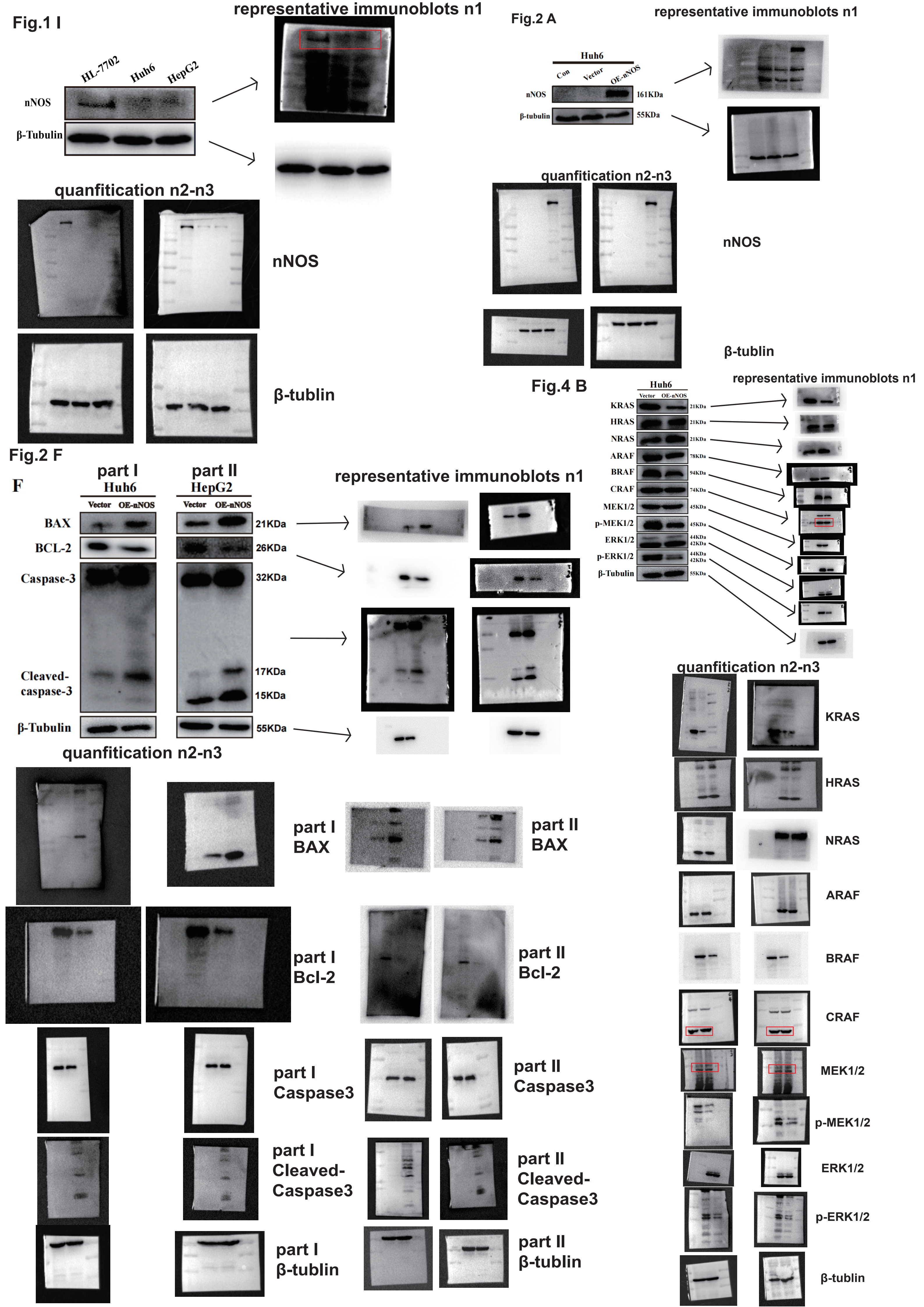
Fig.1 I
representative immunoblots n1
HL-7702
Huh6
HepG2
nNOS
β-Tubulin
quanfitication n2-n3
nNOS
β-tublin
Fig.2 A
representative immunoblots n1
Huh6
Con
Vector
OE-nNOS
nNOS
161KDa
β-tubulin
55KDa
quanfitication n2-n3
nNOS
β-tublin
Fig.4 B
representative immunoblots n1
Huh6
Vector
OE-nNOS
KRAS
21KDa
HRAS
21KDa
NRAS
21KDa
ARAF
78KDa
BRAF
94KDa
CRAF
74KDa
MEK1/2
45KDa
p-MEK1/2
45KDa
ERK1/2
44KDa
42KDa
p-ERK1/2
44KDa
42KDa
β-Tubulin
55KDa
quanfitication n2-n3
KRAS
HRAS
NRAS
ARAF
BRAF
CRAF
MEK1/2
p-MEK1/2
ERK1/2
p-ERK1/2
β-tublin
Fig.2 F
F
part I
Huh6
part II
HepG2
Vector
OE-nNOS
Vector
OE-nNOS
BAX
21KDa
BCL-2
26KDa
Caspase-3
32KDa
Cleaved-caspase-3
17KDa
15KDa
β-Tubulin
55KDa
representative immunoblots n1
quanfitication n2-n3
part I BAX
part II BAX
part I Bcl-2
part II Bcl-2
part I Caspase3
part II Caspase3
part I Cleaved-Caspase3
part II Cleaved-Caspase3
part I β-tublin
part II β-tublin
