# Supplementary figures and images for: nNOS-mediated S-nitrosylation of TCOF1 regulates KRAS proteostasis to suppress hepatoblastoma progression
Source: Redox Biol. 2025 Sep 20;87:103870. doi: 10.1016/j.redox.2025.103870 (PMC12552980; doi:10.1016/j.redox.2025.103870)

**Fig.6 B**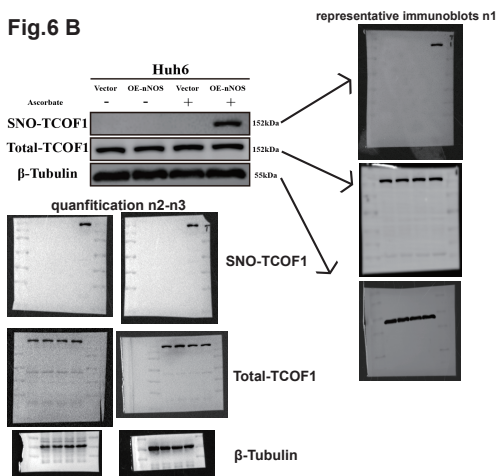**Fig.6 D**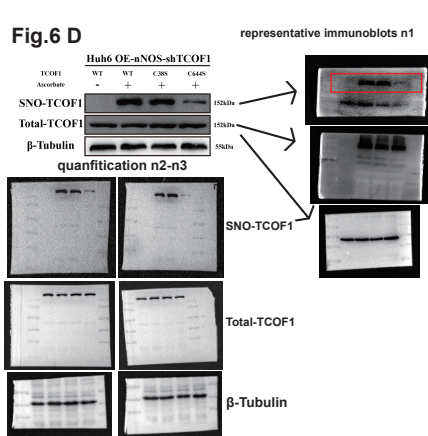**Fig.7 A**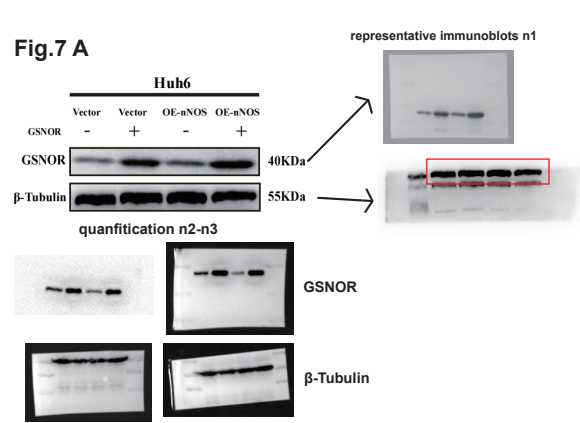**Fig.7 B**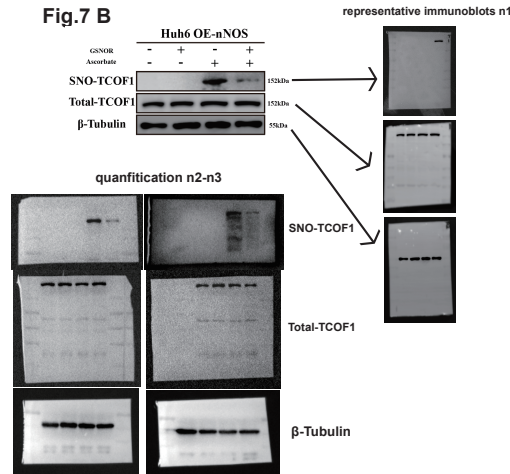**Fig.8 C**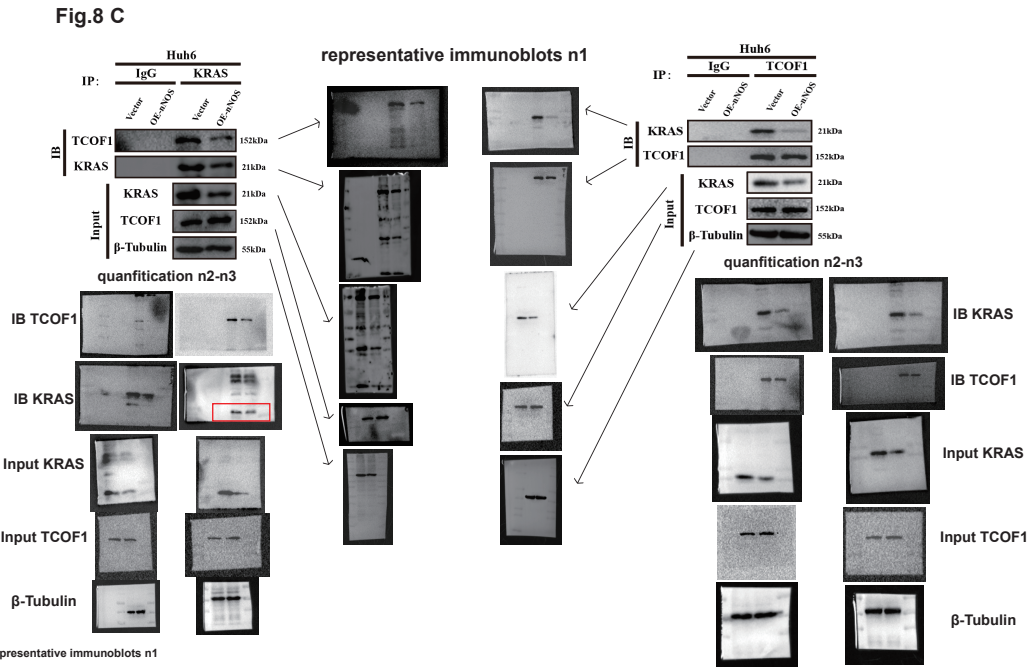**Fig.8 D**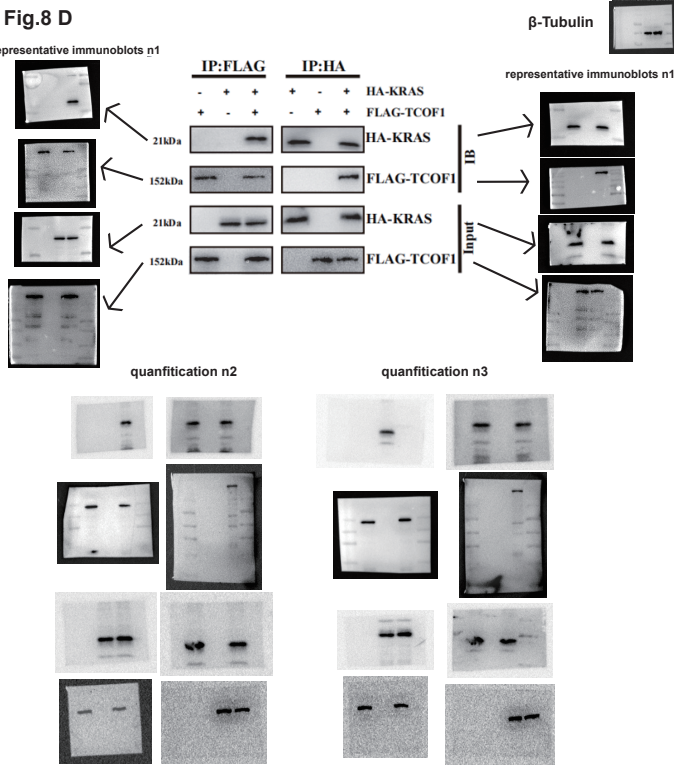**Fig.8 E**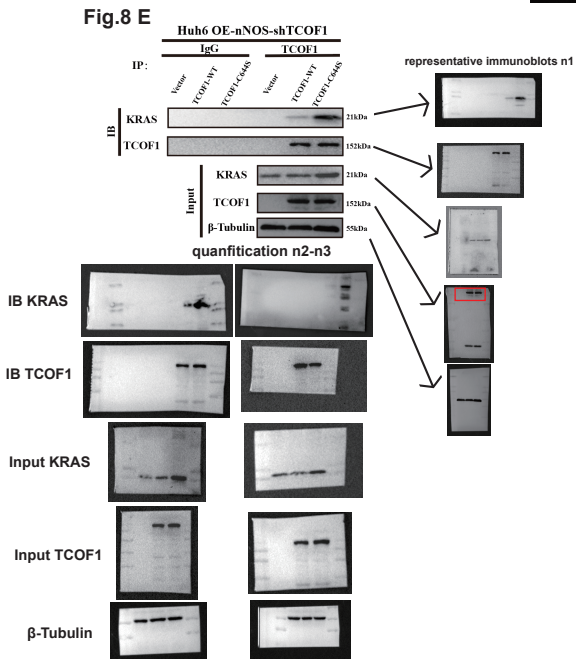**Fig.8 E**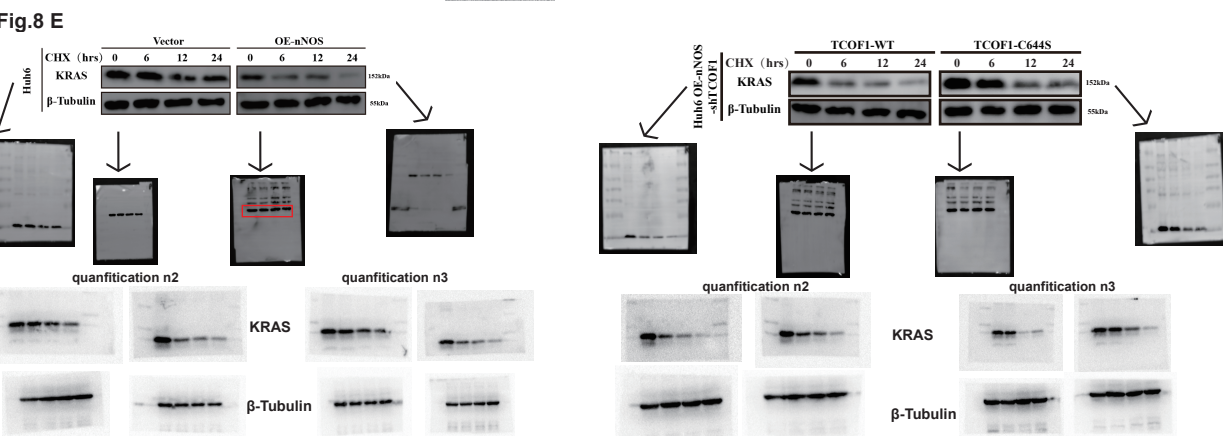

Supplement: Multimedia component 5 [file mmc5.pdf]
